# D–A Type Perylene Micelles With Synergistic Charge/Energy Transfer for Dual‐Path ROS Generation and Enhanced Photocatalysis

**DOI:** 10.1002/advs.76521

**Published:** 2026-07-11

**Authors:** Chenfan Xie, Jin Gao, Guan Wang, Xinyue Huang, Xinyi Hu, Lijun Zhu, Liujun Yang, Hua Li, Jianmei Lu

**Affiliations:** ^1^ College of Chemistry Chemical Engineering and Materials Science Collaborative Innovation Center of Suzhou Nano Science and Technology Soochow University Suzhou P. R. China; ^2^ Suzhou Laboratory Suzhou P. R. China; ^3^ Shaoxing Yucai High School Shaoxing P. R. China; ^4^ State Key Laboratory of Biobased Fiber Manufacturing Technology China Textile Academy Beijing P. R. China

**Keywords:** amphiphilic micelle, charge and energy transfer, molecular oxygen activation, photocatalysis, water purification

## Abstract

Efficient generation of reactive oxygen species (ROS) through synergistic regulation of charge transfer (CT) and energy transfer (ET) pathways is key to enhancing photocatalytic reaction rates. Nevertheless, simultaneously optimizing both singlet (S_1_) and triplet (T_1_) exciton utilization to maximize ROS generation and its subsequent effective exploitation remains challenging. Herein, a series of amphiphilic perylene‐based nanomicelles is designed via donor engineering, in which spatial coupling between dual‐path ROS generation and pollutant oxidation is achieved. Experiments and theoretical calculations certify that the micelle with triphenylamine as the donor can boost the CT efficiency of S_1_ to engender superoxide radical (•O_2_ **^−^**) production, while concurrently improving the intersystem crossing (ISC) efficacy from S_1_ to T_1_ to prolong the T_1_ lifetime and activate the ET process for singlet oxygen (^1^O_2_) yielding. Additionally, the pre‐enrichment of pollutants by micelles shortens the migration distance of ROS and extends their lifetime, thereby maximizing the directed utilization efficiency of ROS. The strategy that combines photophysical properties with structural optimization, successfully surmounts the confinements of organic photocatalytic materials in solar‐driven chemical reactions.

## Graphical abstract

Dual‐state activation of singlet and triplet excitons is achieved in perylene‐based nanomicelles via donor engineering. By synergistically pairing charge transfer with energy transfer pathways, while concurrently enabling pollutant pre‐enrichment, this strategy establishes spatial coupling between efficient ROS generation and rapid mineralization.

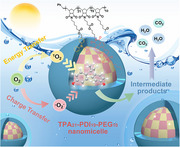

## 1 Introduction

Photocatalytic technology, which harnesses solar energy to drive chemical conversions, is deemed as a pivotal pathway for attaining highly efficient and environmentally friendly redox reactions [1, 2, 3, 4, 5, 6]. Particularly in reaction systems involving ROS, ROS can function as key reactive intermediates and play an essential role in fields such as organic synthesis [7, 8, 9, 10, 11], environmental remediation [12, 13, 14], and energy conversion [15, 16, 17, 18]. Nevertheless, the efficient generation and precise utilization of ROS remains a formidable scientific challenge, which curtails the performance of photocatalytic systems [3, 11, 19].

**FIGURE 1:**
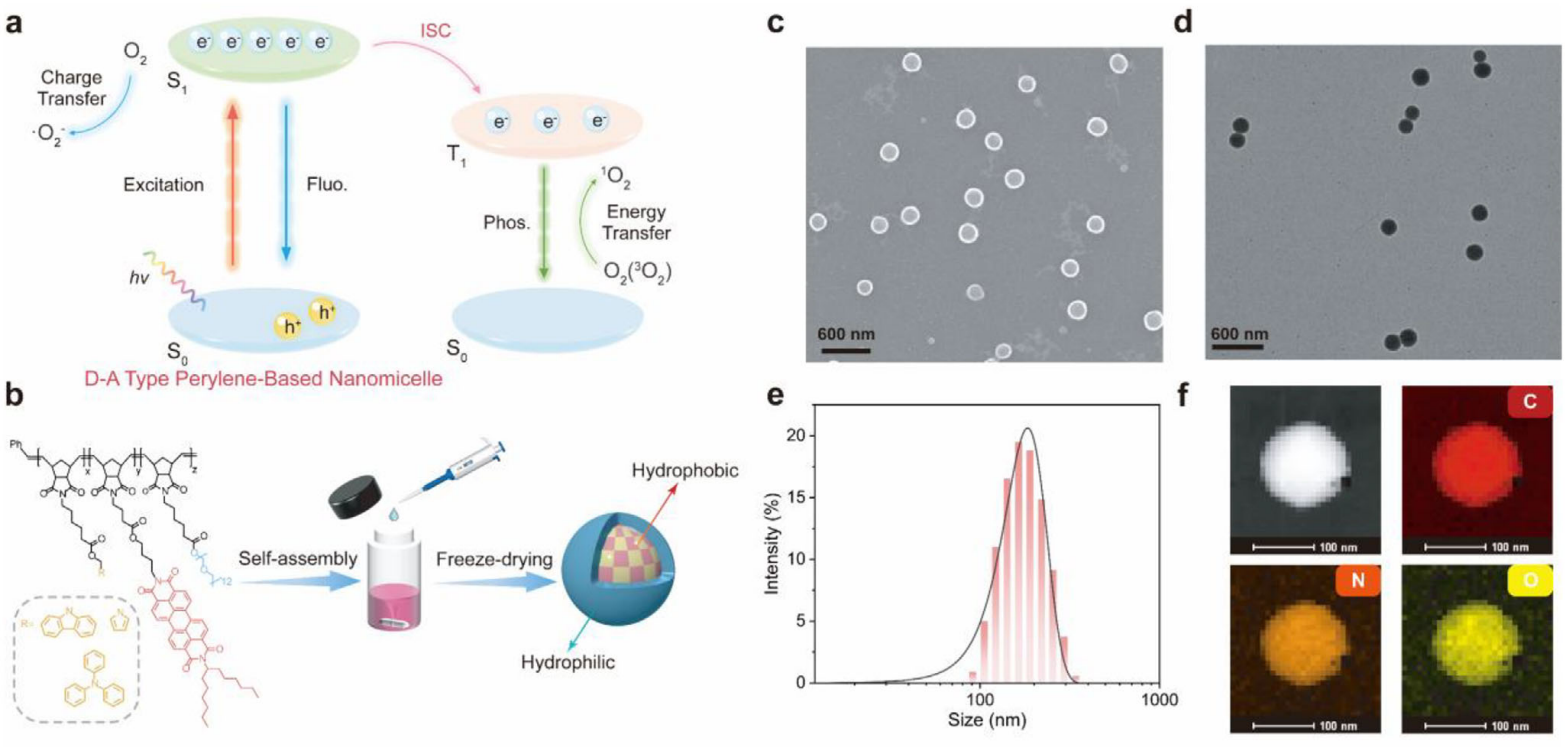
(a)Illustration of CT and ET processes. (b) Schematic preparation of the micelle. (c) SEM, (d) TEM, (e) DLS size distribution, and (f) HAADF‐STEM and elemental distribution images of the TPA_21_‐PDI_19_‐PEG_10_ micelle.

In classical photocatalytic systems (hydrogen evolution [20, 21, 22], H_2_O_2_ synthesis [23, 24, 25], biomass alcohol oxidation [26, 27], etc.), the prevailing mechanism of ROS generation is contingent on the separation of photogenerated charge carriers mediated by direct photon absorption, which populates the S_1_ state. This process generally leverages an augmented CT process to elevate the ROS generation rate, thereby thermodynamically propelling the effective initiation of diverse redox reactions [11, 28, 29, 30, 31]. Notwithstanding many notable strides achieved in the regulation strategies centered on charge separation [32, 33, 34, 35], the efficient generation and targeted deployment of ROS is still impeded by the neglect of ET process, which constitutes another critical pathway for ROS production [15, 36, 37, 38]. By contrast, the ET process hinges on the T_1_ state generated via ISC from the S_1_ state (Figure 1a) [9, 16, 39]. The longevous T_1_ state can effectively circumvent the redox potential constraints inherent in the CT process, facilitating the rapid activation of triplet oxygen (^3^O_2_) to yield ^1^O_2_ while enhancing the stability and catalytic activity of photocatalysts [40, 41]. Based on the aforementioned advantages, the simultaneous optimization of both CT and ET processes is momentous to promoting the efficient generation and maximizing the exploitation of ROS [10, 16, 17, 42, 43, 44].

Specifically, perylene diimide (PDI) and its derivatives, as typical n‐type organic semiconductors and photosensitive materials, exhibit potential for integrating the dual‐state activation strategy within a single system, and have been reported to activate molecular oxygen through both CT and ET pathways [45, 46]. However, it remains imperative to implement tailored design within PDI‐based organic materials to enable optimized management of S_1_ and T_1_ states, and to intensively study the interplay between material structure and dual‐state activation efficiency. Given the electron‐accepting nature of PDI, the fabrication of donor–acceptor (D–A) structures, which allows for precise manipulation of intramolecular charge separation pathways and excited‐state energy migration behaviors, presents a befitting platform for the cocurrent propulsion of dual‐path ROS generation [9, 32, 47, 48]. Furthermore, the targeted utilization efficiency of the generated ROS is also compromised by their ultrashort lifespan and limited diffusion distance in aqueous media [49, 50]. Consequently, for the purpose of facilitating catalytic conversion of reactants, it is essential to accomplish the exquisite spatiotemporal alignment between reactant adsorption and ROS generation sites [14, 51, 52]. Through assembling D–A architectures into amphiphilic micelles, a restricted nanoscale space can feasibly be constructed, which guarantees reactants to be enriched in situ within the ROS generation zone [12, 53]. Such insights are expected to achieve higher photocatalytic efficiency and expanded functional versatility.

In light of the foregoing analysis, a series of D–A polymers, which uses perylene diimide as both the T_1_ state generator and the electron acceptor unit, are synthesized by tuning the donor structure (pyrrole, carbazole, and triphenylamine). The corresponding polymer micelles are then obtained through solvent‐induced self‐assembly (Figure 1b). Theoretical calculations and experimental results enunciate that the triphenylamine (TPA) unit with the strongest electron‐donating ability can effectively harvest both S_1_ and T_1_ excitation energies to drive CT and ET processes, thereby respectively increasing the yields of •O_2_^−^ and ^1^O_2_. In the subsequent application for bisphenol A (BPA) removal, the swift pre‐enrichment of BPA in water by amphiphilic nanomicelles via physical adsorption is inclined to minimize the diffusion distance of ROS, laying the groundwork for ROS targeted exploitation. As a result, TPA_21_‐PDI_19_‐PEG_10_ micelle, which manifests nanoscale spatial coupling between ROS generation and pollutant mineralization, is able to wipe off BPA within 25 min. This strategy of coupling donor‐engineered exciton management with nanomicelle‐induced pollutant pre‐enrichment offers a theoretical approach to effectively enhancing the efficiency of solar‐to‐chemical energy conversion.

## 2 Results and Discussion

### 2.1 Structural and Morphological Characterization

The amphiphilic block polymers are synthesized via ring‐opening metathesis polymerization (ROMP) (Scheme S7). After polymerization, the chemical structure of Block 1, Block 2, and Block 3, which are obtained in the synthesis of polymers is first analyzed by proton nuclear magnetic resonance (^1^H NMR). The appearance of the characteristic peaks that are attributed to different monomers, potently affirms the successful synthesis of three polymers (Figure S1). Gel permeation chromatography (GPC) measurements are then used to determine the molecular weight and molecular weight distribution of the polymers. The number of repeating units per block is calculated based on the GPC results, thus obtaining polymer Py_20_‐PDI_19_‐PEG_10_, Cz_19_‐PDI_18_‐PEG_10,_ and TPA_21_‐PDI_19_‐PEG_10_. The corresponding specific data are listed in Table S1. Finally, the thermal stability of the TPA_21_‐PDI_19_‐PEG_10_ polymer is evaluated by thermogravimetric analysis (TGA). It is displayed in Figure S2 that the pyrolysis temperature of the material at the mass loss of 5% is up to 405°C, which demonstrate the superior thermal stability of the material.

Amphiphilic block copolymers can self‐assemble into homogeneous spherical micelles in deionized water [54]. The morphological changes of the TPA_21_‐PDI_19_‐PEG_10_ polymer before and after self‐assembly are characterized by scanning electron microscopy (SEM) and transmission electron microscopy (TEM) technology. As can be seen from Figure S3, the synthesized polymer displays an irregular morphology with sizes up a few micrometers. Subsequently, under the solvent induction of THF and H_2_O, the polymer TPA_21_‐PDI_19_‐PEG_10_ self‐assembles into micellar spheres with a relatively uniform size of around 200 nm (Figure 1c,d). The particle size distribution of micelles is ulteriorly examined by dynamic light scattering (DLS). It is illustrated in Figure 1e that the hydrodynamic diameter (D_h_) of the micelles is about 213.7 nm, which is consistent with the SEM and TEM results. Meanwhile, the narrow distribution of particle size corroborates the dimensional homogeneity of the micelles. Besides, as shown in Figure 1f, the high‐angle annular dark‐field imaging (HAADF‐STEM) images testify that the elements of C, N, and O are distributed uniformly in the TPA_21_‐PDI_19_‐PEG_10_ micelle. It is postulated that the smaller particle size of the micelles which are constructed through self‐assembly, is conducive to exposing more adsorption sites, thereby immensely facilitating the contacts between the catalyst and the pollutant [55, 56]. Furthermore, the self‐assembly of the TPA_21_‐PDI_19_‐PEG_10_ polymer in water can be revealed by changes in photophysical properties. As is depicted in Figure S4, a bathochromic shift can be clearly observed in the UV–vis absorption spectrum after the formation of micelles, indicating that TPA_21_‐PDI_19_‐PEG_10_ can form J‐aggregates in water after self‐assembly [57]. Therefore, the assembly procedure is likely to minimize the singlet‐triplet energy gap (ΔE_ST_) to facilitate intersystem crossing from S_1_ to T_1_ state by virtue of the aggregation‐induced ISC (AI‐ISC) effect, which is significant to ^1^O_2_ production [58, 59].

### 2.2 Photoelectrochemical Properties Characterization

**FIGURE 2:**
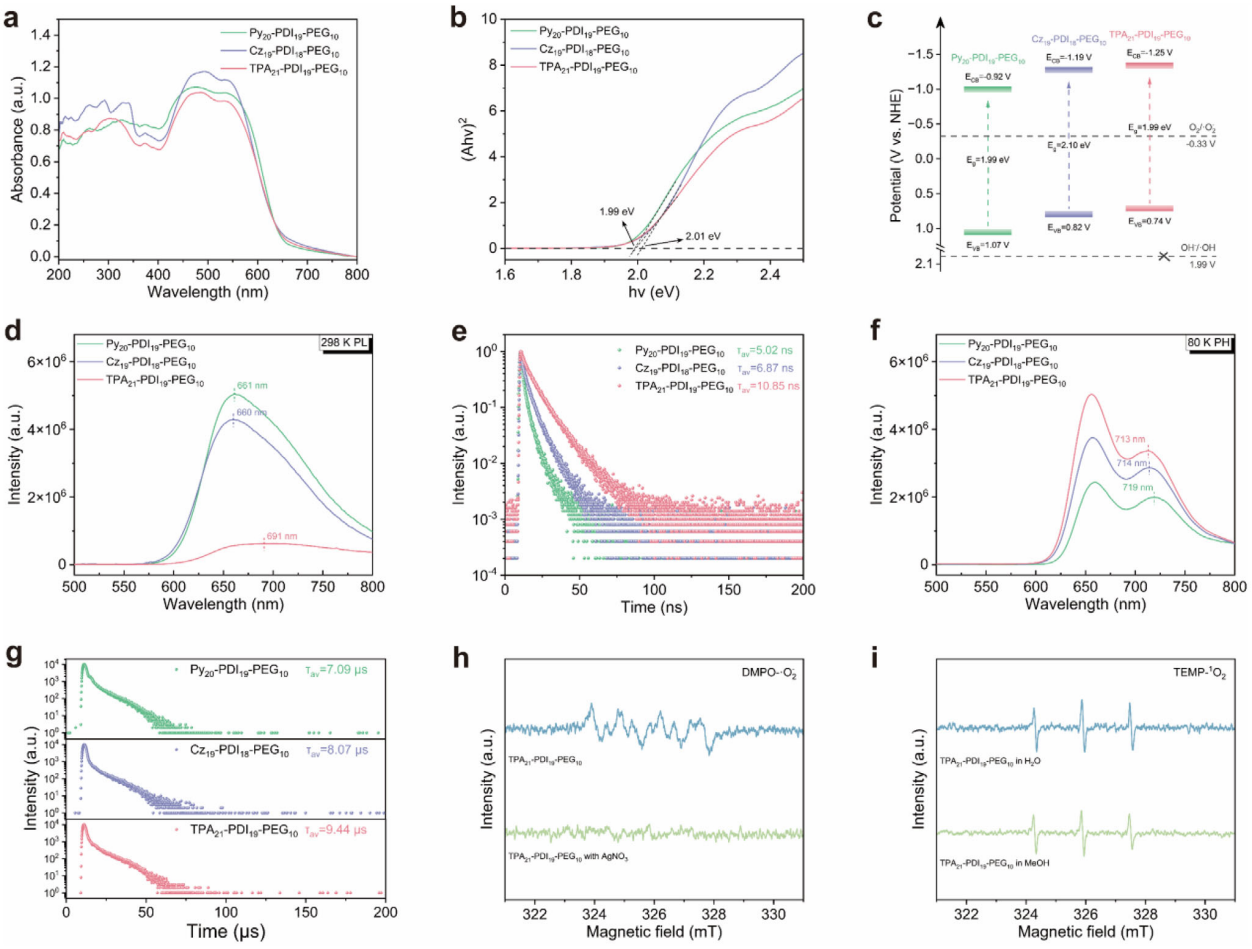
(a) UV–vis diffuse reflectance spectra, (b) Tauc curves, (c) schematic representation of the energy level structure of different micelle samples. Steady/transient‐state PL (d,e) and PH (f,g) spectra of different micelle samples. (h) ESR signal of DMPO‐•O_2_^−^ recorded over different samples in the presence and absence of the e^−^ quencher (AgNO_3_). (i) ESR signal of TEMP‐^1^O_2_ recorded over different samples in the presence and absence of the h^+^ quencher (MeOH).

A multitude of characterizations have been conducted to evaluate the photoelectrochemical properties of the catalysts. First, UV/vis diffuse reflectance spectroscopy (UV/vis DRS) is employed to investigate the bandgaps of the as‐prepared micelles. As shown in Figure 2a, it is evident that all the micelles possess eligible absorption range in visible light region, which may be associated with the exemplary photosensitivity of the PDI unit [8, 60]. Whereafter, the Tauc curves (Figure 2b) are plotted using the Kubelka‐Munk formula, and the bandgaps (E_g_) are calculated to be 1.99, 2.01, and 1.99 eV for Py_20_‐PDI_19_‐PEG_10_, Cz_19_‐PDI_18_‐PEG_10_, and TPA_21_‐PDI_19_‐PEG_10_, respectively. Next, the conduction band (E_CB_) levels of different catalysts are measured by Mott–Schottky plots. As is presented in Figure S5, all three micelles are n‐type semiconductors, and the E_CB_ positions of Cz_19_‐PDI_18_‐PEG_10_, Py_20_‐PDI_19_‐PEG_10,_ and TPA_21_‐PDI_19_‐PEG_10_ amount to −1.19, −0.92, and −1.25 V (vs standard hydrogen electrode (NHE)), respectively. Furthermore, the valence band (E_VB_) levels of Cz_19_‐PDI_18_‐PEG_10_, Py_20_‐PDI_19_‐PEG_10,_ and TPA_21_‐PDI_19_‐PEG_10_ are calculated as 0.82, 1.07, and 0.74 V (vs. NHE). The energy level distributions of the three photocatalysts are demonstrated in Figure 2c, and the corresponding data of the energy band structure is listed in Table S2. Notably, the energy level structure of the TPA_21_‐PDI_19_‐PEG_10_ micelle shifts upward relative to other catalysts, so that the reduction ability of photogenerated electrons can get more powerful, which is in favor of the efficient reduction of O_2_. Besides, comparing to Cz_19_‐PDI_18_‐PEG_10_ and Py_20_‐PDI_19_‐PEG_10_, the TPA_21_‐PDI_19_‐PEG_10_ photocatalyst exhibits a better photocurrent response as well as a smaller impedance radius (Figure S6), which furnishes compelling evidence for the outstanding CT capability of the TPA_21_‐PDI_19_‐PEG_10_ micelle [61].

More importantly, in an effort to comprehensively elucidate the enhancement of CT and ET processes in the catalysts, steady/transient‐state photoluminescence (PL)/phosphorescence (PH) spectra excited at 375 nm are recorded. It is illustrated in Figure 2d that the fluorescence intensity of micelles adheres to the following order: Py_20_‐PDI_19_‐PEG_10_> Cz_19_‐PDI_18_‐PEG_10_> TPA_21_‐PDI_19_‐PEG_10_. The most pronounced fluorescence quenching in TPA_21_‐PDI_19_‐PEG_10_ is indicative of the most efficient separation of electrons and holes, thus enhancing CT efficiency [62]. Simultaneously, the PL peak of TPA_21_‐PDI_19_‐PEG_10_ redshifts to around 691 nm, corresponding to the decreased energy gap for the S_1_ state. Moreover, the time‐resolved PL decay profiles in Figure 2e show biexponential decay behavior for all micelles. The fast decay component (τ_1_), assigned to singlet‐state radiative recombination, exhibited a duration of 1.50 ns for Py_20_‐PDI_19_‐PEG_10_, 2.33 ns for Cz_19_‐PDI_18_‐PEG_10_, and 4.11 ns for TPA_21_‐PDI_19_‐PEG_10_, contributing 57.94%, 40.11%, and 25.92% to the overall decay, respectively. Conversely, the long‐lived component (τ_2_), pertaining to charge‐separated states, accounted for 42.06% for Py_20_‐PDI_19_‐PEG_10_, 59.89% for Cz_19_‐PDI_18_‐PEG_10_, and 74.08% for TPA_21_‐PDI_19_‐PEG_10_, suggesting that as the electron‐donating capacity of the donor increases, the excited‐state lifetime of materials is prolonged (Table S3). This extended lifetime signifies suppressed electron–hole recombination and enhanced electron transfer efficiency [63]. Additionally, the fully charge‐separated excited state derived from the introduction of the D–A structure is liable to increase the probability of T_1_ formation [8, 17]. What's more, TPA_21_‐PDI_19_‐PEG_10_ exhibits augmented PH emission intensity (Figure 2f), implying the boosted T_1_ exciton yield, which is often credited to the promoted ISC efficiency arising from the decreased energy gap between S_1_ and T_1_ excited states (ΔE_ST_) [64]. By comparing the energy separations between the PL and PH peaks [13, 65], the ΔE_ST_ values of Cz_19_‐PDI_18_‐PEG_10_, Py_20_‐PDI_19_‐PEG_10_, and TPA_21_‐PDI_19_‐PEG_10_ are calculated to be 0.14, 0.16, and 0.06 eV, respectively. The considerably lower ΔE_ST_ signifies that TPA_21_‐PDI_19_‐PEG_10_ is more liable to productively stimulate the transition of excitons from the S_1_ to the T_1_ state via the ISC process [15]. In addition, the prolonged PH lifetime of TPA_21_‐PDI_19_‐PEG_10_ is of benefit to increase the probability of its collision with the ground state oxygen (^3^O_2_), therefore promoting the generation of ^1^O_2_ via the ET process [16] (Figure 2f and Table S4). Finally, it can be concluded from the above outcomes that TPA_21_‐PDI_19_‐PEG_10_ successfully enables the dual‐path reinforcement of both CT and ET processes.

### 2.3 ROS Generation Evaluation and Theoretical Explanation

Above all, to elucidate the formation pathways of •O_2_^−^ and ^1^O_2_, electron spin resonance (ESR) trapping‐tests are performed in the presence of e^−^ quencher (AgNO_3_) and h^+^ quencher (MeOH). Evidently, the DMPO‐•O_2_^−^ signal disappears in the presence of AgNO_3_ (Figure 2h), clarifying that the production of •O_2_^−^ is in compliance with the CT process. Since the E_CB_ values of all micelles are negative than the standard electrode potential of O_2_/•O_2_^−^ (Figure 2c), the reduction of O_2_ to form •O_2_^−^ through the CT pathway is feasible [66]. In CT process, D–A micelles are excited by illumination to transform from the ground state (S_0_) to the S_1_ state via photon transition (hν), inducing photogenerated electrons to flow from the HOMO energy level to the LUMO energy level, where the absorbed O_2_ is reduced to •O_2_^−^ (Figure 1a). Furthermore, as is displayed in Figure 2i, the TEMP‐^1^O_2_ signal of TPA_21_‐PDI_19_‐PEG_10_ is well preserved even in MeOH, signifying that the transformation of ^1^O_2_ does not depend on the CT process, but derives from the ET process from the micelle to oxygen. The PDI unit with outstanding photosensitive activity can serve as T_1_ state generator, and subsequently deliver energy to ^3^O_2_. ^3^O_2_ contains two unpaired electrons with the same spin direction in its outer orbital, and it can interact with the exited photosensitizer to result in the inversion of the spin of one of the unpaired electrons, thus obtaining the more reactive ^1^O_2_.

**FIGURE 3:**
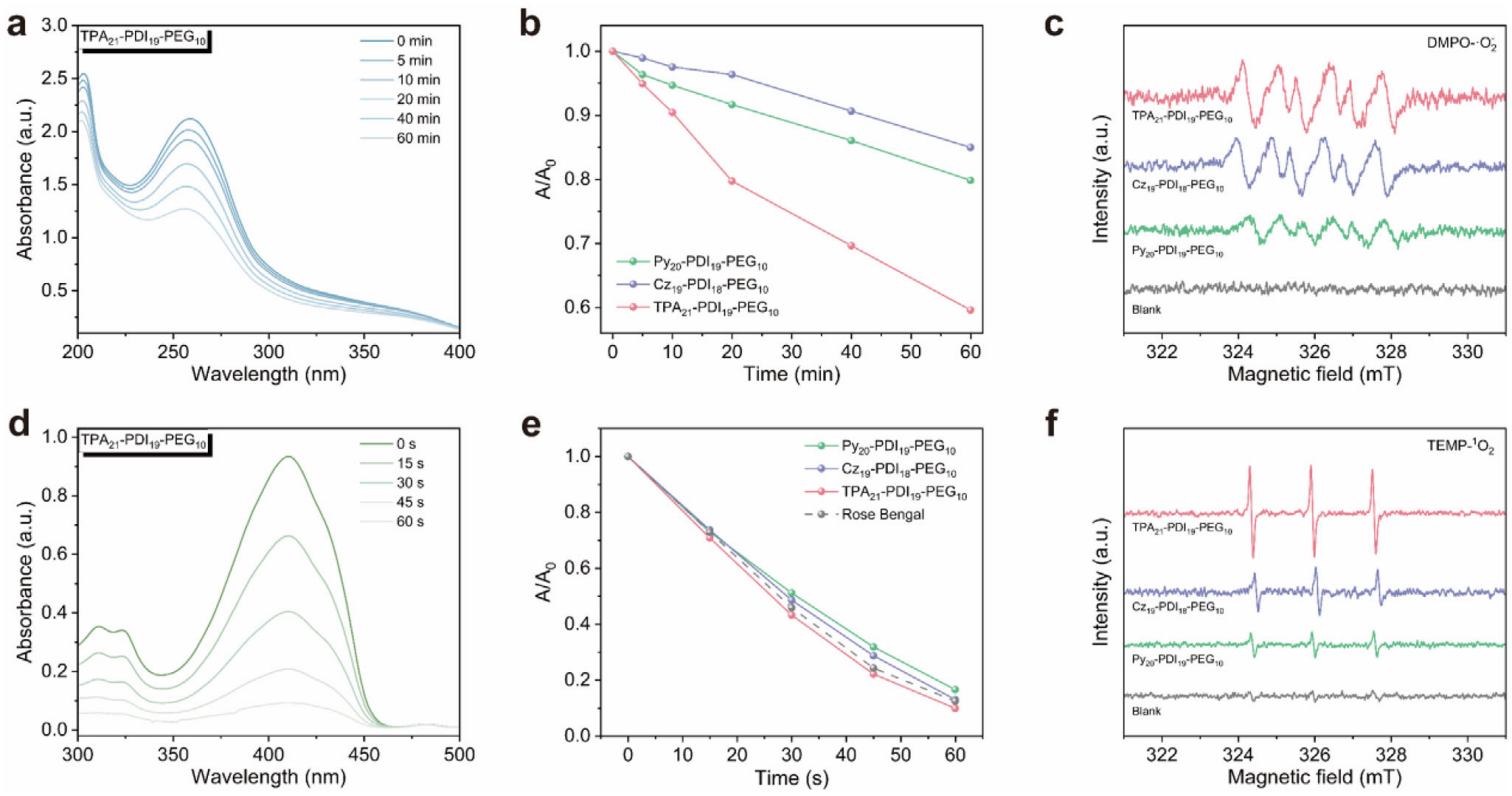
(a) Time‐dependent UV/vis absorption spectra of NBT in deionized water upon visible light irradiation (λ > 420 nm) in the presence of TPA_21_‐PDI_19_‐PEG_10_ micelle. (b) The absorbance of NBT oxidation with different micelles. (c) ESR signal of DMPO‐•O_2_^−^ recorded over different micelles. (d) Time‐dependent UV/vis absorption spectra of DPBF in MeCN upon light irradiation (λ  = 550 nm) in the presence of TPA_21_‐PDI_19_‐PEG_10_ micelle. (e) The absorbance of DPBF oxidation with different micelles. (f) ESR signal of TEMP‐^1^O_2_ recorded over different micelles.

For the sake of evaluating the ROS generation capability of different micelles, fluorescence probe experiments and ESR measurements are carried out. As for the generation of •O_2_^−^, nitrotetrazolium blue chloride (NBT) is employed as the probe molecule to determine the capacity of •O_2_^−^ generation (Figure 3a,b and Figure S8a,b). The concentrations of •O_2_^−^ generated by Py_20_‐PDI_19_‐PEG_10_, Cz_19_‐PDI_18_‐PEG_10_ and TPA_21_‐PDI_19_‐PEG_10_ after 60 min visible light irradiation reach 22.49, 29.16 and 62.46 µmol L^−1^, respectively (Figure S8d and Table S5). Besides, using 5,5‐dimethyl‐1‐pyrroline N‐oxide (DMPO) as the trapping agent, all the micelles exhibit DMPO‐•O_2_^−^ signals, with TPA_21_‐PDI_19_‐PEG_10_ showing a relatively stronger signal consistent with the NBT absorption spectrum trend (Figure 3c). The prominently higher yield of •O_2_^−^ by TPA_21_‐PDI_19_‐PEG_10_ can be attributed to the introduction of the powerful donor unit (TPA), which yields preferable separation of photogenerated charge carriers, thereby rendering the photoinduced electron transfer exceptionally rapid and efficient [10, 67, 68].

With regard to ^1^O_2_, degradation assays using 1,3‐diphenylisobenzofuran (DPBF) are conducted. TPA_21_‐PDI_19_‐PEG_10_ micelle exhibits a sharp descent in DPBF absorbance to almost zero within 60 s, whereas Cz_19_‐PDI_18_‐PEG_10_ and Py_20_‐PDI_19_‐PEG_10_ show a lower efficacy (Figure 3d,e and Figure S9c–e). Then, the ^1^O_2_ generation quantum yield is measured with rose Bengal (RB) as the reference. As a result, the singlet oxygen quantum yield (Φ_Δ_)of TPA_21_‐PDI_19_‐PEG_10_ is calculated as 0.59, which is superior to that of Cz_19_‐PDI_18_‐PEG_10_ (0.51) and Py_20_‐PDI_19_‐PEG_10_ (0.45) (Figure S9f and Table S6). Subsequently, the ESR technique is utilized to further verify the efficiency of ^1^O_2_ production, with 2,2,6,6‐tetramethylpiperidine (TEMP) as the trapping agent (Figure 3f). The most distinct triplet signal of TEMP‐^1^O_2_ adducts eloquently validates that TPA_21_‐PDI_19_‐PEG_10_ micelle can efficiently generate ^1^O_2_ under light illumination. The narrowing of ΔE_ST_ by effectively separating the HOMO and LUMO is conducive to elevating ISC efficiency and accelerating ET process to form ^1^O_2_ [69, 70]. The ΔE_ST_ for different micelles follows the order: Py_20_‐PDI_19_‐PEG_10_< Cz_19_‐PDI_18_‐PEG_10_< TPA_21_‐PDI_19_‐PEG_10_, aligning with the capacity of ^1^O_2_ generation by different micelles. In addition, through comparing the ^1^O_2_ concentrations of TPA_21_‐PDI_19_‐PEG_10_ before and after self‐assembly, it can be reasonably deduced that intermolecular aggregation induced by the self‐assembly process are inclined to improve ISC efficiency, resulting in an enhancement in ^1^O_2_ production.

**FIGURE 4:**
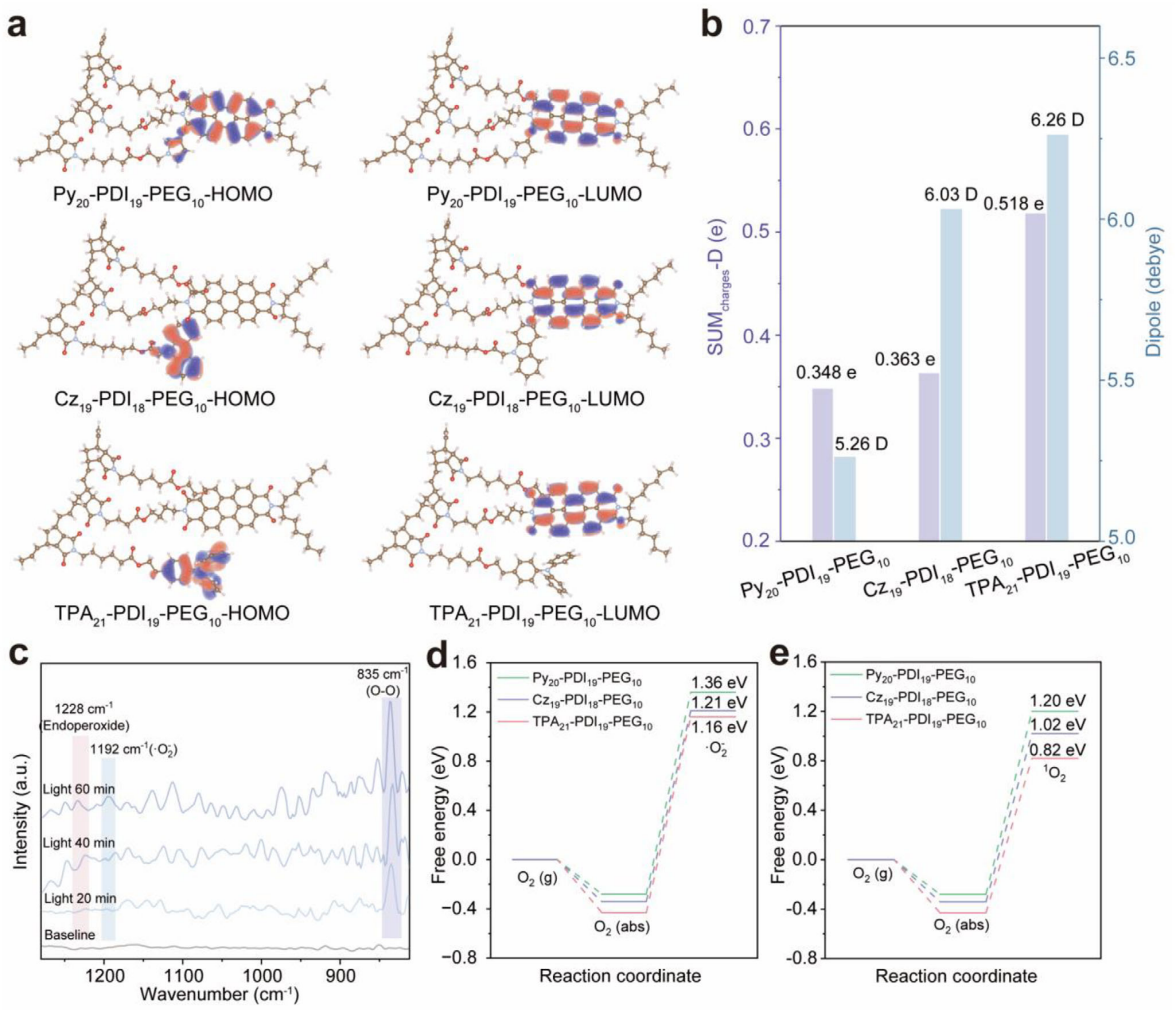
(a) HOMO and LUMO distribution in Py_20_‐PDI_19_‐PEG_10_, Cz_19_‐PDI_18_‐PEG_10_ and TPA_21_‐PDI_19_‐PEG_10_ polymers. (b) Mulliken atomic charges and dipole moments of Py_20_‐PDI_19_‐PEG_10_, Cz_19_‐PDI_18_‐PEG_10,_ and TPA_21_‐PDI_19_‐PEG_10_ polymers (c) In situ DRIFTS of TPA_21_‐PDI_19_‐PEG_10_ micelle under O_2_. Gibbs free energy diagrams of (d) CT and (e) and ET pathways for O_2_ conversion over TPA_21_‐PDI_19_‐PEG_10_ micelle.

Density functional theory (DFT) calculations are performed to gain profound insights into the ROS generation mechanism. First, the Mulliken atomic charges of each polymer are calculated (Figure S11). Since the sum of the charges of all atoms in each polymer structure is 0.00 e, the electron‐donating capacity of different donors can be quantified in terms of the sum of the charges of all the donor units in each polymer (SUM_charges_‐D) [71, 72]. The SUM_charges_‐D values of Py_20_‐PDI_19_‐PEG_10_, Cz_19_‐PDI_18_‐PEG_10_ and TPA_21_‐PDI_19_‐PEG_10_ are calculated to be 0.348, 0.363, and 0.518 e, respectively (Figure 4b). The highest SUM_charges_‐D value of TPA_21_‐PDI_19_‐PEG_10_ demonstrates the strongest the electron‐donating capacity of the TPA donor in the polymer structure. Thereafter, the HOMO (highest occupied molecular orbital)/LUMO (lowest unoccupied molecular orbital) energy levels of three fabricated polymers are investigated. As is revealed in Figure 4a, TPA_21_‐PDI_19_‐PEG_10_ displays the greatest degree of electron cloud delocalization, where HOMO is mainly located at the TPA unit, while LUMO is uniformly distributed over the PDI unit. Furthermore, the calculated dipole moments of Py_20_‐PDI_19_‐PEG_10_, Cz_19_‐PDI_18_‐PEG_10_, and TPA_21_‐PDI_19_‐PEG_10_ are 5.26, 6.03, and 6.26 D, respectively (Figure 4b). It is particularly noteworthy that systems with large dipole moments exhibit pronounced spatial separation between HOMO and LUMO. Such spatial separation, not only elicits distinctive CT character via enhanced charge carrier separation efficiency, but also shrinks ΔE_ST_, which is favorable for the occurrence of ISC [7]. It can be summarized that the construction of D–A architecture integrates dual‐path reinforcement of both CT and ET processes, thereby enabling simultaneous amplification in the production of •O_2_^−^ and ^1^O_2_.

Additionally, in order to support the formation of •O_2_^−^ (CT pathway) and ^1^O_2_ (ET pathway), key reaction intermediates are identified by performing in situ diffuse reflectance infrared Fourier transform spectroscopy (DRIFTS) over the TPA_21_‐PDI_19_‐PEG_10_ micelle under an oxygen and steam environment. Noticeably, as is recorded in Figure 4c, signals corresponding to endoperoxide (1228 cm^−1^), •O_2_^−^ (1192 cm^−1^) and O−O (835 cm^−1^) gradually intensify with irradiation time, which serves as direct evidence for the involvement of both CT and ET pathways in the photo‐driven ROS generation process [10, 16]. Gibbs free energy calculations are then performed to evaluate dual pathways for O_2_ conversion (Figure 4d,e). The O_2_ adsorption energies of Py_20_‐PDI_19_‐PEG_10_, Cz_19_‐PDI_18_‐PEG_10_, and TPA_21_‐PDI_19_‐PEG_10_ are calculated as −0.34, −0.28, and −0.43 eV, respectively. The lowest energy barrier indicates that TPA_21_‐PDI_19_‐PEG_10_ is more preferable for the adsorption and activation of O_2_, thus facilitating the subsequent light‐driven conversion. For the CT pathway via S_1_ as well as the ET pathway via T_1_, calculations also identify that TPA_21_‐PDI_19_‐PEG_10_ hosts the lowest Gibbs energy to form •O_2_^−^ and ^1^O_2_, which interprets its highest ROS yields from a thermodynamic perspective.

### 2.4 Photocatalytic Activity Investigation and Degradation Pathway Analysis

In order to evaluate the photocatalytic performance of the photocatalysts, BPA (50 mg L^−1^) is selected as the target pollutant. Above all, it is universally acknowledged that adsorption plays a dominant role under light‐free conditions. As is presented in Figure S12a, all three polymer micelles, which are able to reach adsorption equilibrium within 10 s, displayed exceptional adsorption performance. Simultaneously, there is no significant difference in the adsorption ratios of BPA. The BPA adsorption ratios of Cz_19_‐PDI_18_‐PEG_10_, Py_20_‐PDI_19_‐PEG_10_, and TPA_21_‐PDI_19_‐PEG_10_ are 65.2%, 63.2%, and 66.1%, respectively. The excellent adsorption capability mentioned above can be rationally ascribed to the *π*–*π* interactions between bisphenol A and the conjugated structure of the micelles, as well as the hydrogen bonding formed between the phenolic hydroxyl group and the carbonyl oxygen atom of the PDI unit [55, 81]. Moreover, the hydrophilic segment of the polymers allows the micelles to be well dispersed in water, while the hydrophobic interactions between the hydrophobic core and BPA further promote the rapid capture of BPA by the micelles [55]. The detailed discussion of adsorption performance and mechanisms are given in the Supporting Information. Thanks to the highly efficient enrichment of BPA by micelles, it is facile for the ROS generated during the subsequent catalytic process to exert swift and accurate effects on pollutant molecules, which can overcome the constraint of the short lifespan of ROS and increase the overall mineralization efficiency.

**FIGURE 5:**
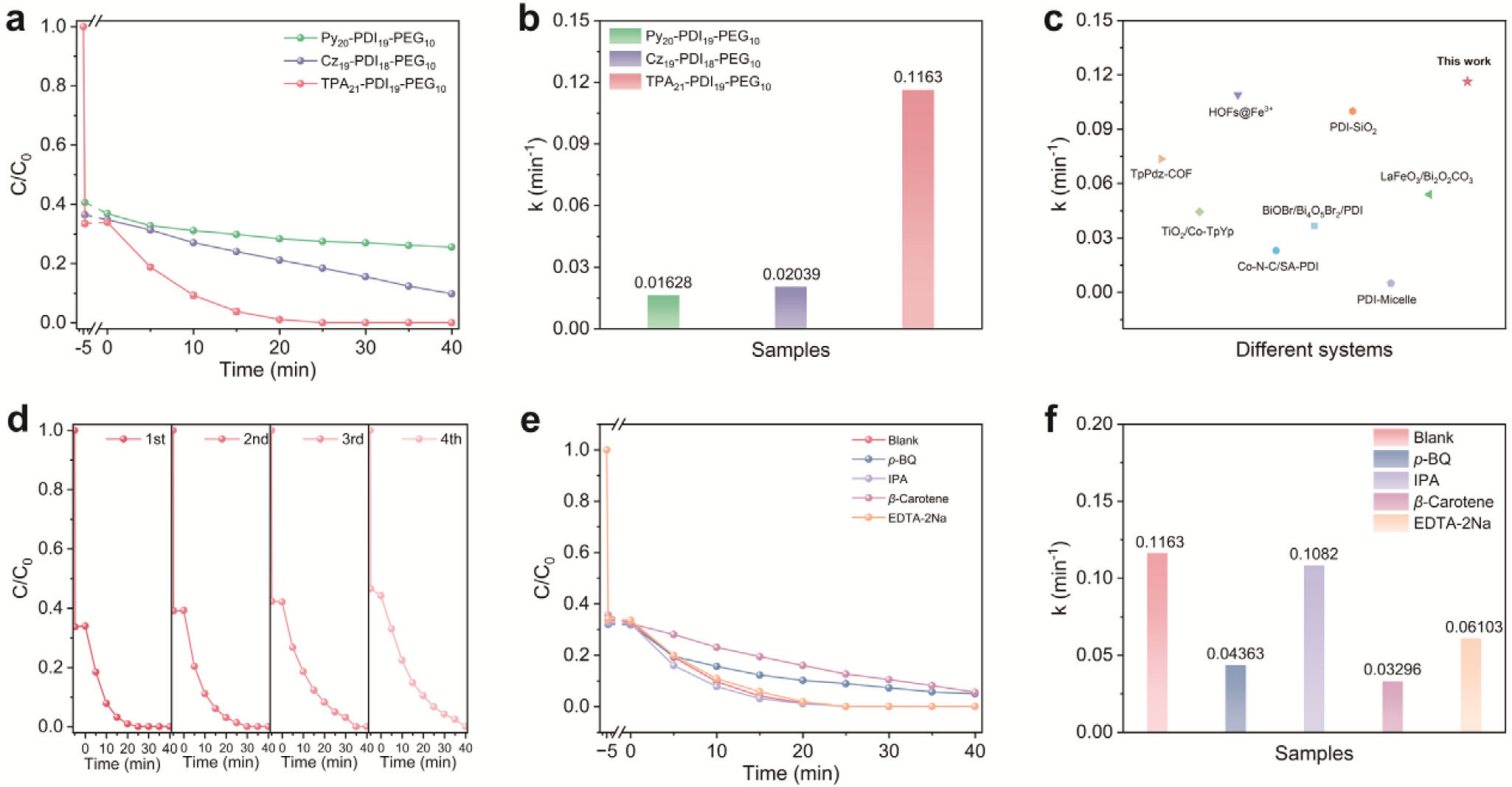
(a) Photodegradation curves of BPA over different micelles. (b) Degradation kinetic rate constants of BPA over different micelles. (c) Comparison of kinetic rate constants (k) for BPA degradation with other reported photocatalysts [55, 73, 74, 75, 76, 77, 78, 79, 80]. (d) Cyclic adsorption and photodegradation of the TPA_21_‐PDI_19_‐PEG_10_ micelle to BPA. (e) Photodegradation curves and (f) degradation rate constants of BPA by TPA_21_‐PDI_19_‐PEG_10_ micelle in the presence of different scavengers.

Next, the photodegradation performance of BPA under visible light irradiation is delineated in Figure 5a. The degradation performance of Py_20_‐PDI_19_‐PEG_10_ and Cz_19_‐PDI_18_‐PEG_10_ micelles is less than satisfactory, with the BPA removal ratios of 90.2% and 74.4% at 40 min, respectively. In comparison, TPA_21_‐PDI_19_‐PEG_10_ showcases the best degradation performance, in the presence of which the BPA existing in aqueous solution can be absolutely removed within just 25 min. So as to further inquire into the degradation condition of BPA inside the micelles, methanol is utilized as the desorption solvent for the pre‐extracted catalyst samples. It is illustrated in Figure S12b that the TPA_21_‐PDI_19_‐PEG_10_ micelle exhibits the optimal degradation of BPA, with only 6.49% of BPA remaining inside the micelles at 25 min, which can be completely removed at 40 min. The degradation curves of BPA are then fitted to a pseudo‐first‐order kinetic model (Figure S12c), and the reaction rate constants (k) are calculated accordingly (Figure 5b). The TPA_21_‐PDI_19_‐PEG_10_ micelle shows the highest reaction rate (0.1163 min^−1^), which is respectively 7.14 and 5.70 times higher than Py_20_‐PDI_19_‐PEG_10_ (0.01628 min^−1^) and Cz_19_‐PDI_18_‐PEG_10_ (0.02039 min^−1^). Meanwhile, the k value of TPA_21_‐PDI_19_‐PEG_10_ is superior to that of the currently reported photocatalysts made of pure organic materials, and even surpasses many metal‐based photocatalytic materials (Figure 5c). It is apparent that the photodegradation activity of the catalysts coincides with their generation capacity of •O_2_^−^ and ^1^O_2_, suggesting the significant involvement of ROS in degradation process.

What's more, cycling expected are conducted to appraise the reusability of the TPA_21_‐PDI_19_‐PEG_10_ micelle (Figure 5d). After 4 cycles, the micelle can still entirely remove BPA from the water within 40 min, which powerfully proves the recyclability of the catalyst in removing BPA. Additionally, the chemical stability of the catalyst is also explored. From the ^1^H NMR spectra of the polymer micelles before and after photocatalysis (Figure S17), it can be observed that the characteristic peaks of the monomers TPA, PDI, and PEG do not show obvious variation. SEM, TEM, and DLS tests are further performed to manifest that the micelle can still maintain its spherical morphology with a virtually unchanged D_h_ value (226.2 nm) after photocatalysis (Figures S18 and S19). These tests jointly indicate the extraordinary structural stability of the micelle. Besides, aiming at elaborating the practical application value of the TPA_21_‐PDI_19_‐PEG_10_ micelle, the catalytic degradation performance of the micelle is tested in various water qualities. As is depicted in Figure S20a, the coexisting of ubiquitous inorganic anions (Cl^−^, NO_3_^−^, H_2_PO_4_^−^, and HCO_3_^−^) exerts negligible influence on the overall contaminant removal efficiency. Impressively, BPA can be totally removed within 25 min in real water matrices such as tap water, river water, and lake water (Figure S20b), which further testifies to the favorable environmental tolerance of the micelle. Next, the removal ability of the micelle for different types of pollutants is measured as well. The TPA_21_‐PDI_19_‐PEG_10_ micelle exhibits moderate catalytic activity for TCP, DCP, 4‐chlorophenol, p‐cresol, and methyl orange (Figure S20c), certifying the modest removal capability for certain types of contaminants of the micelle.

To clarify photodegradation pathways of BPA by TPA_21_‐PDI_19_‐PEG_10_ micelle, free radical trapping experiments are performed. As is revealed in Figure 5e, after the addition of IPA, there is no obvious change in the degradation rate of BPA, signifying that there is almost no •OH involved in the photocatalytic reaction of the TPA_21_‐PDI_19_‐PEG_10_ micelle. The presence of EDTA‐2Na slightly impedes the photocatalytic activity, with a k value of 0.06103 min^−1^, while the addition of p‐BQ and β‐carotene leads to considerable suppression, with the k values decreasing to 0.04363 and 0.03296 min^−1^, respectively (Figure 5f and Figure S21). Hence, it can be presumed that ^1^O_2_ and •O_2_^−^ play a major part in the photodegradation of BPA, while h^+^ is served as a minor factor, and the contribution degree is ^1^O_2_> •O_2_^−^ > h^+^. Noticeably, the contribution of ^1^O_2_ to photocatalysis is far more significant than that of h^+^, which supports the view that ^1^O_2_ is primarily generated via the direct ET process rather than the oxidation of •O_2_^−^ by photo‐generated holes. Subsequently, liquid chromatography‐mass spectrometry (LC‐MS) is exploited to detect the degradation intermediates of BPA in the TPA_21_‐PDI_19_‐PEG_10_ micelle. It is depicted in Figure S22 that there are eight types of intermediates detected. Based on these findings, a possible degradation process of BPA is proposed rationally (Figure S23). First, the exquisite spatiotemporal coupling between pollutant capture and ROS production guarantees maximal harnessing of short‐lived ROS. Then under the synergistic action of ^1^O_2_, •O_2_^−^, and h^+^, BPA is gradually degraded into a variety of small molecules, which can be utterly broken down to CO_2_ and H_2_O in the end of the photocatalytic process.

In addition, to assess the toxicity of the degradation intermediates, the toxicity of BPA and the intermediates are predicted using Ecological Structure Activity Relationships (ECOSAR) software. According to the corresponding analysis results, the acute toxicity and chronic toxicity of all intermediates are lower than that of the initial pollutant, confirming the significant detoxification of the degradation process (Figure S24). Ecotoxicity assays using wheat seeds as research subjects further validate the potential for effectively eliminating BPA pollution and mitigating ecological toxicity in aquatic environments (Figure S25). Moreover, total organic carbon (TOC) analysis reveals mineralization efficiency of 90.62%, demonstrating remarkable mineralization capacity of TPA_21_‐PDI_19_‐PEG_10_ micelle (Figure S26). As such, it can be concluded that the TPA_21_‐PDI_19_‐PEG_10_ micelle can not only effectively eliminate and mineralize water contaminants, but also conspicuously decrease their toxicity, thus holding considerable potential for environmentally sustainable contaminant remediation.

## 3 Conclusion

In summary, we have prepared a series of D–A type perylene‐based polymer micelles, where dual‐state activation can be integrated through donor‐optimization engineering to deliver efficient ROS generation. Particularly, the nanomicelle which features TPA as the electron donor, is able to concurrently amplify the effective harnessing of both S_1_ and T_1_ excitation energies, and expedite the efficient generation of •O_2_^−^ and ^1^O_2_. Besides, the nanoscale micelle can enable rapid enrichment of BPA, which substantially improves the targeted utilization of ROS, thereby accomplishing the photodegradation removal of BPA within a short period of time. This strategy of integrating S_1_ and T_1_ photoexcitation energies into a unified photocatalytic material, might present a viable theoretical framework for the advancement of more efficient and multifunctional photocatalytic systems.

## Author Contributions

**Jin Gao**: methodology, conceptualization, formal analysis. **Guan Wang**: formal analysis. **Chenfan Xie**: conceptualization, methodology, software, writing – review and editing, writing – original draft, formal analysis, supervision, project administration. **Jianmei Lu**: formal analysis, project administration, resources, writing – review and editing, funding acquisition. **Liujun Yang**: formal analysis. **Lijun Zhu**: formal analysis. **Hua Li**: formal analysis, funding acquisition, writing – review and editing, resources, supervision, project administration. **Xinyue Huang**: formal analysis. **Xinyi Hu**: formal analysis.

## Funding

This work is financially supported by the NSF of China (22438009, 22278285, U24A20535), Science and Technology Program of Jiangsu (BK20243002), Science and Technology Program of Suzhou (2022SS20) and Priority Academic Program Development of Jiangsu Higher Education Institutions (PAPD).

## Conflicts of Interest

The authors declare no conflicts of interest.

## Supporting information




**Supporting File**: advs76521‐sup‐0001‐SuppMat.pdf.

## Data Availability

The data that support the findings of this study are available from the corresponding author upon reasonable request.
