# Supplementary material for: D–A Type Perylene Micelles With Synergistic Charge/Energy Transfer for Dual‐Path ROS Generation and Enhanced Photocatalysis
Source: Adv Sci (Weinh). 2026 Jul 11:e76521. Online ahead of print. doi: 10.1002/advs.76521 (PMC13355907; doi:10.1002/advs.76521)
Supplement: Supplementary file 1 — Supporting File: advs76521‐sup‐0001‐SuppMat.pdf. [file ADVS-9999-e76521-s001.pdf]

D-A Type Perylene Micelles with Synergistic Charge/Energy Transfer for Dual-Path ROS Generation and Enhanced Photocatalysis

Chenfan Xie[1,2], Jin Gao[3], Guan Wang[4], Xinyue Huang[1,2], Xinyi Hu[1,2], Lijun Zhu[1,2], Liujun Yang[1,2,], Hua Li[1,2,]*, Jianmei Lu[1,2,]**

Experimental section

Chemicals

Grubbs' second-generation catalyst (Grubbs' 2nd cat.) is purchased from ALDRICH cooperation. 9H-Carbazole, cis-5-norbornene-exo-2,3-dicarboxylic anhydride, pivalic anhydride, 4-aminobutanoic acid, 6-aminocaproic acid, ammonium acetate (NH_4OAc), 4-amino-1-butanol, β-carotene, p-chlorophenol, and p-cresolare purchased from Aladdin. Bisphenol A (BPA) is bought from Alfa Aesar company. Perylene-3,4,9,10-tetracarboxylic dianhydride, polyethylene glycol monomethyl ether (550), 4-dimethylaminopyeidine (DMAP), imidazole, 7-tridecanon, 1-(2-hydroxyethyl) pyrrole, and p-benzoquinone (BQ) are purchased from TCI. Sodium cyanoborohydride ($NaBH_3CN$), *t*-butanol (*t*-BuOH), and isopropanol (IPA) are produced by Macklin. Ethylenediaminetetraacetic acid disodium salt (EDTA-2Na) is bought from Sinopharm Chemical Reagent Co., Ltd.. 4-Diphenylaminobenzyl alcohol is purchased from Leyan Chemistry.

Synthesis of copolymers

Synthesis of 4-((4R,7S)-1,3-dioxo-1,3,3a,4,7,7a-hexahydro-2H-4,7-methanoisoindol-2-yl)butanoic acid (Compound 1)

cis-5-norbornene-exo-2,3-dicarboxylic anhydride (6.00 g, 36.5 mmol), 4-aminobutyric acid (3.58 g, 34.8 mmol) and 250 mL of toluene were added to 500 mL three-necked bottle equipped with a water-oil separator. The reaction was refluxed at 140 °C for 24 h under N_2 atmosphere. After the reaction was completed, most of the toluene was removed by rotary evaporation, and then the residue was dissolved in 200 mL CH_2Cl_2, followed by washing with 1 M HCl and saturated NaCl solution. The organic phase was dried over anhydrous Na_2SO_4 to remove residual water, and the obtained organic phase was filtered and evaporated to dryness to give a pale brown solid (7.37 g, 85%). ^{1}H NMR (400 MHz, DMSO-d_6) δ 12.10 (s, 1H), 6.30 (d, J = 1.9 Hz, 2H), 3.38 (t, J = 7.0 Hz, 2H), 3.09 (s, 2H), 2.68 (d, J = 1.4 Hz, 2H), 2.20 (t, J = 7.4 Hz, 2H), 1.67 (p, J = 7.3 Hz, 2H), 1.37 (dp, J = 9.8, 1.7 Hz, 1H), 1.11 (dt, J = 9.7, 1.5 Hz, 1H).

Synthesis of tridecan-7-amine (Compound 2)

7-tridecanone (20 g, 100.86 mmol), NH_4OAc (77.46 g, 1004.97 mmol), $NaBH_3CN$ (4.53 g, 72.09 mmol mmol) and MeOH (240 mL) were added to a 500 mL round-bottom flask, and the mixture stirred at room temperature (r.t.) for 48 h. Subsequently, the reaction was quenched by dropwise addition of 16 mL aqueous HCl (2 M), and the MeOH was removed by rotary evaporation. The obtained white solid was redissolved in H_2O then adjusted to pH=10 with solid KOH. The compound was extracted with CH_2Cl_2 and dried by anhydrous Na_2SO_4, which was concentrated to give a pale yellow oil (16.9 g, 84%).

Synthesis of 2,9-di(tridecan-7-yl)anthra[2,1,9-def:6,5,10-d'e'f']diisoquinoline-1,3,8,10(2H,9H)-tetraone (Compound 3)

Perylene-3,4,9,10-tetracarboxylic dianhydride (3.9 g, 9.97 mmol), compound 2 (4.77 g, 23.92 mmol) and imidazole (15.9 g) were added in a 100 mL round-bottom flask. The reaction was refluxed at 160 °C for 5 h. After the mixture was cooled to r.t., it was dissolved with 400 mL EtOH and treated with 400 mL aqueous HCl (2 M), stirred overnight. The dark red precipitate was filtered and dried in vacuo at 130 °C to give a dark red powder (6.89 g, 91%).^{1}H NMR (400 MHz, Chloroform-*d*) δ 8.80 - 8.52 (m, 8H), 5.18 (tt, J = 7.4, 5.8 Hz, 2H), 2.25 (dt, J = 14.1, 9.7 Hz, 4H), 1.85 (dt, J = 9.9, 3.4 Hz, 4H), 1.33-1.20 (m, 32H), 0.82 (t. J = 6.8 Hz, 12H).

Synthesis of 9-(tridecan-7-yl)-1H-isochromeno[6',5',4':10,5,6]anthra[2,1,9-def] isoquinoline-1,3,8,10(9H)-tetraone (Compound 4)

Compound 3 (6.89 g, 9.16 mmol), KOH (1.71 g, 30.63 mmol) and 168 mL t-BuOH were added in a 500 mL round-bottom flask. The reaction was refluxed at 90 °C for 30 min. After the mixture was cooled to r.t., it was treated with 80 mL acetic acid and 40 mL aqueous HCl (2 M), stirred overnight. The dark red precipitate was filtered, washed with deionized water and dried in vacuo at 130 °C to give a dark red solid (3.35 g, 64%).^{1}H NMR (400 MHz, Chloroform-*d*) δ 8.94 - 8.36 (m, 8H), 5.18 (tt, J = 9.4, 5.8 Hz, 1H), 2.24 (dtd, J = 14.2, 9.7, 4.8 Hz, 2H), 1.96 - 1.80 (m, 2H), 1.33 (s, 4H), 1.25 - 1.18 (m, 12H), 0.82 (t, J = 6.7 Hz, 6H).

Synthesis of 2-(4-hydroxybutyl)-9-(tridecan-7-yl)anthra[2,1,9-def:6,5,10-d'e'f'] diisoquinoline-1,3,8,10(2H,9H)-tetraone (Compound 5)

Compound 4 (3.35 g, 5.82 mmol), 4-amino-1-butanol (0.793 g, 8.899 mmol) and imidazole (12.84 g) were added in a 500 mL round-bottom flask, the reaction was refluxed at 180 °C for 5 h. After the mixture was cooled to r.t., it was dissolved with 150 mL EtOH and treated with 300 mL aqueous HCl (2 M), stirred overnight. The dark red precipitate was filtered and dried in vacuo at 130 °C to give a dark red solid (3.42 g, 91%).^{1}H NMR (400 MHz, Chloroform-*d*) δ 8.68 - 8.38 (m, 8H), 5.01 (s, 1H), 4.22 (d, J = 7.5 Hz, 1H), 3.78 (t, J = 6.3 Hz, 2H), 3.46 (s, 2H),

2.25 (dt, J = 14.0, 9.7 Hz, 2H), 1.89 (s, 2H), 1.79 (p, J = 6.5 Hz, 2H), 1.44 (s, 2H), 1.37 - 1.26 (m, 4H), 1.26 - 1.19 (m, 12H), 0.87 - 0.76 (m, 6H).

Synthesis of PDI monomer (Monomer 1)

Compound 1 (1.528 g, 6.14 mmol), Compound 5 (3.29 g, 5.11 mmol), pivalic anhydride (1.14 mg, 6.14 mmol) and DMAP (62.43 mg, 0.511 mmol) and 247 mL of THF were separately added to a 500 mL single-necked flask. The reaction was refluxed at 70 °C for 24 h. After the reaction was completed, 1 mL deionized water was added and stirred for 1 h, then washed with saturated $NaHCO_3$ solution and saturated NaCl solution, anhydrous Na_2SO_4 was added to the organic phase and filtered out, and the filtrate was dried by rotary evaporator. The product was further purified by silica gel column chromatography (eluent: ethyl acetate/n-hexane=3/1, v/v) to give a dark red solid (3.25 g, 78%). ^{1}H NMR (400 MHz, Chloroform-d) δ 8.71 - 8.48 (m, 8H), 6.26 (t, J = 1.9 Hz, 2H), 5.18 (tt, J = 9.3, 5.8 Hz, 1H), 4.28 - 4.13 (m, 4H), 3.52 (t, J = 7.1 Hz, 2H), 3.24 (p, J = 1.7 Hz, 2H), 2.65 (d, J = 1.3 Hz, 2H), 2.37 - 2.18 (m, 4H), 1.90 - 1.76 (m, 4H), 1.58 (s, 4H), 1.54 - 1.45 (m, 1H), 1.32 (s, 4H), 1.28 - 1.16 (m, 12H), 0.90 - 0.76 (m, 6H).

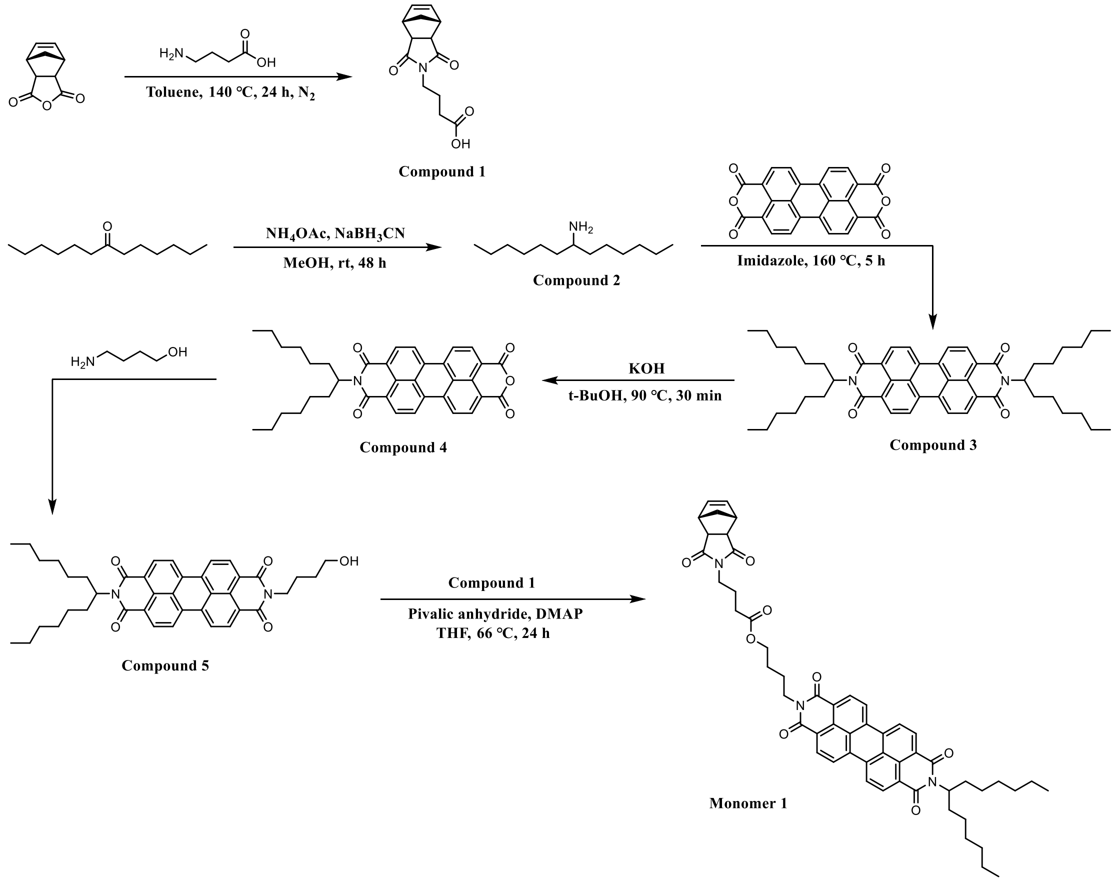

Scheme S1. Synthesis procedure of PDI monomer (Monomer 1)[1].

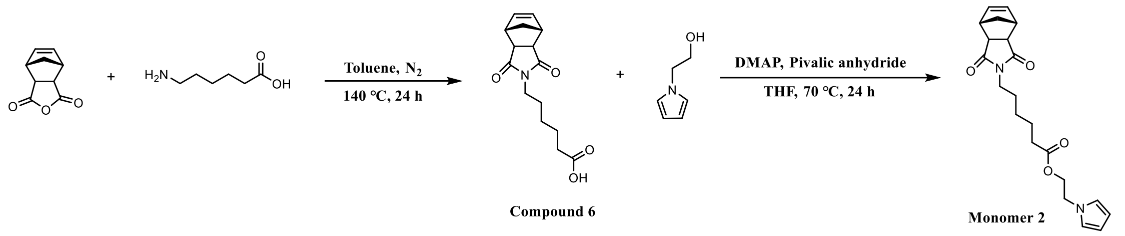

Scheme S2. Synthesis procedure of Py monomer (Monomer 2).

Synthesis of 6-((4R,7S)-1,3-dioxo-1,3,3a,4,7,7a-hexahydro-2H-4,7-methanoisoindol-2-yl)hexanoic acid (Compound 6)

cis-5-norbornene-exo-2,3-dicarboxylic anhydride (4.00 g, 24.4 mmol) and 6-aminocaproic acid (3.19 g, 24.3 mmol) and 250 mL of toluene were separately added to a 500 mL three-necked flask equipped with a water-oil separator. The mixture was refluxed at 140 °C for 24 h under N_2 atmosphere. After completion of the reaction, the toluene was spun dry, dissolved in CH_2Cl_2, and washed with a 1 M HCl solution and a saturated sodium chloride solution. The organic phase was dried over anhydrous Na_2SO_4 to remove residual moisture. The obtained organic phase was dried using a rotary evaporator to give a pale yellow oil (7.43 g, 88 %). ^{1}H NMR (400 MHz, DMSO-d_6) δ 11.99 (s, 1H), 6.30 (s, 2H), 3.32 (d, J = 7.1 Hz, 2H), 3.09 (s, 2H), 2.68 (d, J = 1.4 Hz, 2H), 2.17 (t, J =7 .3 Hz, 2H), 1.56 - 1.38 (m, 4H), 1.38 - 1.33 (m, 1H), 1.23 (tdd, J = 8.7, 6.3, 1.9 Hz, 2H), 1.11 (d, J = 9.7 Hz, 1H).

Synthesis of Py monomer (Monomer 2)

Compound 6 (1.499 g, 5.408 mmol), 1-(2-hydroxyethyl)pyrrole (500 mg, 4.50 mmol), pivalic anhydride (1.007 g, 5.408 mmol), DMAP (54.95 mg, 0.450 mmol) and THF (200 mL) were added to a 500 mL flask. The reaction was refluxed at 70 °C for 24 h. After the reaction was completed, 1 mL of deionized water was added and stirred for 1 h, and then washed successively with saturated $NaHCO_3$ and NaCl solution. Anhydrous Na_2SO_4 was added to the organic phase to remove residual water, and after filtration, the filtrate was dried by a rotary evaporator to obtain a pale-yellow oil. The sample was further purified by silica gel column chromatography (eluent: dichloromethane/ethyl acetate = 90/10, v/v) to give a colorless oil (1.23 g, 74%).^{1}H NMR (400 MHz, DMSO-d_6) δ 6.75 (t, J = 2.1 Hz, 2H), 6.31 (t, J = 1.8 Hz, 2H), 5.98 (t, J = 2.1 Hz, 2H), 4.24 (t, J = 5.3 Hz, 2H), 4.11 (t, J = 5.3 Hz, 2H), 3.31 (d, J = 7.2 Hz, 2H), 3.10 (t, J = 1.9 Hz, 2H), 2.68 (d, J = 1.3 Hz, 2H), 2.26 (t, J = 7.3 Hz, 2H), 1.55 - 1.32 (m, 6H), 1.29 - 1.08 (m, 2H). ^{13}C NMR (101 MHz, DMSO-d_6) δ 178.09, 172.94, 138.09, 121.28, 108.22, 64.22, 47.95, 47.70, 44.91, 42.81, 38.14, 33.66, 27.31, 26.14, 24.32.

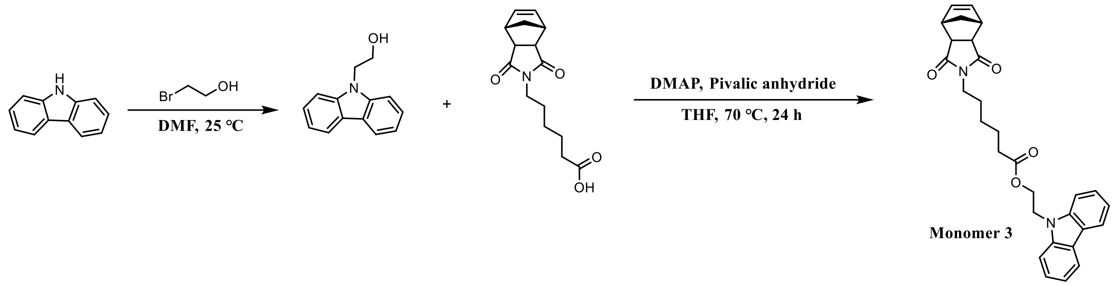

Scheme S3. Synthesis procedure of Cz monomer (Monomer 3)[2].

Synthesis of 2-(9H-carbazol-9-yl)ethan-1-ol (Compound 7)

9H-carbazole (6.69 g, 40.06 mmol), KOH (14.53 g, 259.05 mmol) and DMF (70 mL) were added to a 250 mL round-bottomed flask and the reaction was carried out at 25 °C for 1.5 h. Then 2-bromo-1-ethanol (7.74 g, 61.98 mmol) dissolved in 10 mL of DMF solution was added dropwise to the flask in 1 h. The reaction was continued for another 3 h and poured into 1 L deionized water and left for 24 h. Subsequently, the above solution was filtered, followed by dissolving the white cottony solid in a mixed solution (ethanol: water = 3 : 7) and filtering to remove the insoluble solid. Finally, 500 mL of deionized water was injected into the filtrate and a cottony white solid appeared after recrystallization, which was filtered and dried in vacuo to give a white solid (3.86 g, 45%). ^{1}H NMR (400 MHz, DMSO-d_6) δ 8.14 (dt, *J* = 7.8, 1.0 Hz, 2H), 7.60 (dt, *J* = 8.2, 0.9 Hz, 2H), 7.50 - 7.35 (m, 2H), 7.23 - 7.16 (m, 2H), 4.87 (t, *J* = 5.5 Hz, 1H), 4.43 (t, *J* = 5.8 Hz, 2H), 3.78 (q, *J* = 5.7 Hz, 2H).

Synthesis of Cz monomer (Monomer 3)

Compound 6 (3.16 g, 11.39 mmol), compound 7 (2.00 g, 9.47 mmol), pivalic anhydride (1.14 g, 6.14 mmol), DMAP (62.43 mg, 0.511 mmol) and 247 mL THF were separately added to a 500 mL single-necked flask. The reaction was refluxed at 70 °C for 24 h. After the reaction was completed, 1 mL of deionized water was added and stirred for 1 h, and then washed successively with saturated $NaHCO_3$ and NaCl solution. Anhydrous Na_2SO_4 was added to the organic phase to remove residual water, and after filtration, the filtrate was dried by a rotary evaporator to obtain a pale-yellow oil. The sample was further purified by silica gel column chromatography (eluent: dichloromethane/methanol=5/1, v/v) to give a pale yellow solid (3.62 g, 81%). ^{1}H NMR (400 MHz, DMSO-d_6) δ 8.13 (dt, *J* = 7.8, 0.9 Hz, 2H), 7.64 - 7.40 (m, 4H), 7.19 (td, *J* = 7.5, 0.9 Hz, 2H), 6.30 (t, *J* = 1.9 Hz, 2H), 4.66 (t, *J* = 5.3 Hz, 2H), 4.41 (t, *J* = 5.3 Hz, 2H), 3.32 (s, 2H), 3.22 (t, *J* = 7.3 Hz, 2H), 2.66 (d, *J* = 1.3 Hz, 2H), 2.01 (t, *J* = 7.3 Hz, 2H), 1.42 - 1.16 (m, 5H), 1.13 - 0.90 (m, 3H). ^{13}C NMR (101 MHz, DMSO-d_6) δ 178.05, 172.87, 140.57, 138.08, 126.08, 122.63, 120.67, 119.36, 109.80, 62.44, 47.67, 44.91, 42.79, 41.81, 38.07, 33.61, 27.23,

26.02, 24.02. Calcd for: C, 74.02%; H, 6.43%; N, 5.95%. Found: C, 74.07%; H, 6.29%; N, 5.73%.

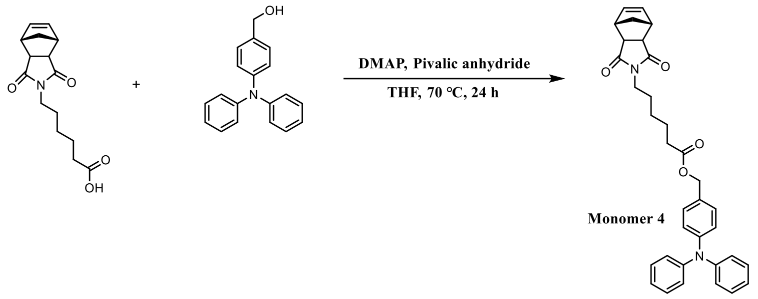

Scheme S4. Synthesis procedure of TPA monomer (Monomer 4).

Synthesis of TPA monomer (Monomer 4)

Compound 6 (543.48 mg, 2.183 mmol), 4-Dimethylaminobenzyl alcohol (500 mg, 1.816 mmol), pivalic anhydride (407 mg, 2.186 mmol), DMAP (22.19 mg, 0.182 mmol) and 80.86 mL THF were separately added to a 250 mL single-necked flask. The reaction was refluxed at 70 °C for 24 h. After the reaction was completed, 1 mL of deionized water was added and stirred for 0.5 h, and then washed successively with saturated $NaHCO_3$ and NaCl solution. Anhydrous Na_2SO_4 was added to the organic phase to remove residual water, and after filtration, the filtrate was dried by a rotary evaporator to obtain a pale-yellow oil. The sample was further purified by silica gel column chromatography (eluent: n-hexane/ethyl Acetate =15/355, v/v) to give a pale yellow oil (875 g, 90%). ^{1}H NMR (400 MHz, DMSO-d_6) δ 7.14 - 7.00 (m, 8H), 6.92 - 6.83 (m, 6H), 6.12 (t, J = 1.9 Hz, 2H), 4.87 (s, 2H), 3.33 - 3.23 (m, 2H), 3.10 (t, J = 1.8 Hz, 2H),2.18 (t, J = 7.5 Hz, 2H), 1.55 - 1.31 (m, 5H), 1.12 - 1.07 (m, 3H). ^{13}C NMR (101 MHz, DMSO-d_6) δ 178.08, 173.15, 147.79, 147.54, 138.08, 130.66, 130.26, 130.01, 124.42, 123.88, 123.53, 65.57, 47.69, 44.90, 38.15, 33.70, 27.30, 26.19, 24.42.

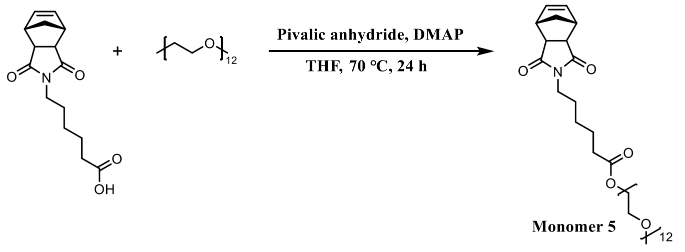

Scheme S5. Synthesis procedure of PEG monomer (Monomer 5)[1].

Synthesis of PEG monomer (Monomer 5)

Compound 6 (3.03 g, 10.9 mmol), polyethylene glycol monomethyl ether 550 (5 g, 9.1 mmol), pivalic anhydride (2.03 g, 10.9 mmol), DMAP (0.11 g, 0.91 mmol) and 120 mL THF were separately added to a 250 mL single-necked flask. The reaction was refluxed at 70 °C for 24 h. After the reaction was completed, 1 mL of deionized water was added and stirred for 0.5 h, and then washed successively with saturated $NaHCO_3$ and NaCl solution. Anhydrous Na_2SO_4 was

added to the organic phase to remove residual water, and after filtration, the filtrate was dried by a rotary evaporator to obtain a pale-yellow oil. The sample was further purified by silica gel column chromatography (eluent: dichloromethane/methanol=95/5, v/v) to give the pure product (4.64 g, 63%). ^{1}H NMR (400 MHz, DMSO-d_6) δ 6.30 (t, J = 1.8 Hz, 2H), 4.15 - 4.07 (m, 2H), 3.63 - 3.55 (m, 2H), 3.51 (s, 46H), 3.46 - 3.39 (m, 2H), 3.24 (s, 3H), 3.09 (p, J = 1.7 Hz, 2H), 2.68 (d, J = 1.4 Hz, 2H), 2.28 (t, J = 7.3Hz, 2H), 1.58-1.49 (m, 2H), 1.44 (dt, J = 15.0, 7.4 Hz, 2H), 1.37 (dt, J = 9.7, 1.7 Hz, 1H), 1.23 (tt, J = 9.9, 6.1 Hz, 2H), 1.16-1.07 (m, 3H). ^{13}C NMR (101 MHz, DMSO-d_6) δ 178.09, 173.20, 138.09, 70.25, 63.54, 58.51, 47.69, 44.91, 42.81, 33.64, 27.32, 26.18, 24.39.

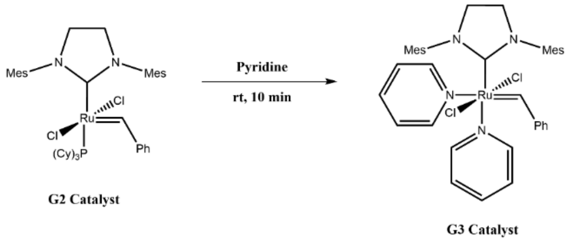

Scheme S6. Synthesis procedure of G3 catalyst[1].

Synthesis of Grubbs' 3rd Generation Catalyst (G3)

Grubbs' 2nd cat. (100 mg, 0.12 mmol) and pyridine (508 mg, 6.43 mmol) were added to a 50 mL vial, and stirred at 28 °C for 10 min. When the solution became clearly green, 30 mL pentane was added to precipitate the product, after cooled in refrigerator for 12 h, the green G3 was filtered, and washed three times with cold pentane and dries in vacuo at 40 °C to obtain a pure green solid (72.6 mg, 86%).

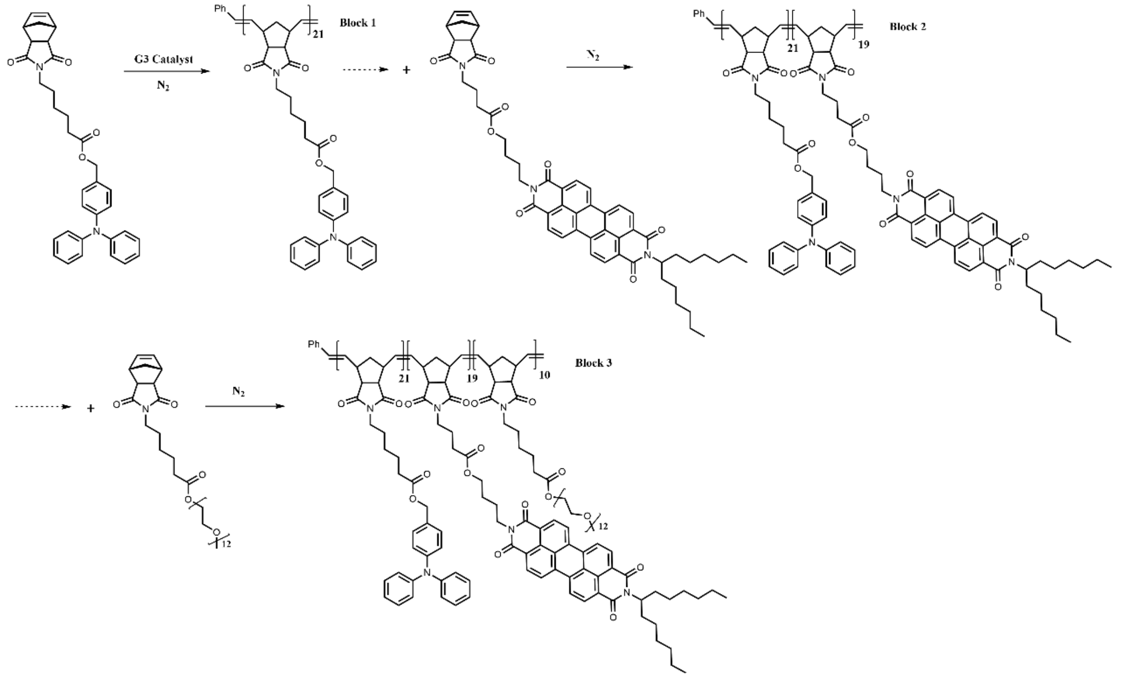

Scheme S7. Synthesis procedure of the TPA_{21}-PDI_{19}-PEG_{10} polymer.

Synthesis of TPA_{21}-PDI_{19}-PEG_{10} Copolymer

Under N_2 atmosphere, G3 (8.3 mg, 0.0114 mmol) was dissolved by 2 mL CH_2Cl_2 in a 20 mL vial. TPA monomer (122.2 mg, 0.229 mmol), PDI monomer (200 mg, 0.229 mmol), and PEG monomer (92.4 mg, 0.114 mmol) were dissolved in 2 mL CH_2Cl_2 under the same condition. Then the solution of monomer was rapidly injected into the catalyst solution to start polymerization, the reaction was stirred at 28 °C for 1 h in N_2 atmosphere. One hour later, 0.3 mL of the reaction solution was withdrawn and terminated with ethylvinyl ether (0.5 mL) for further analysis of the first block of the triblock copolymer (block 1). Subsequently, the solution of PDI monomer was added to the polymerization, and reacted for another 1 h. Similarly, after one hour, 0.3 mL of the reaction solution was withdrawn and terminated with ethylvinyl ether (0.5 mL) for further analysis of the first two blocks of the triblock copolymer (block 2). Finally, the solution of PEG monomer was added to the polymerization, and reacted for another 1 h. At the end of the reaction, 1 mL of vinyl ether was added and stirred for another 0.5 h to terminate the polymerization. The block 1 and block 12 solution were poured into 40 mL cold diethyl ether respectively, then centrifuged and dried to obtain polymer block 1 and block 12. The final reaction solution was poured into 40 mL cold diethyl ether, then centrifuged and dried. The polymer was further purified by dichloromethane dissolution-cold diethyl ether precipitation-centrifugation operation and dried under vacuum to obtain a pure amphiphilic triblock copolymer TPA_{21}-PDI_{19}-PEG_{10} (block 3, 312.6 mg, 75%).

Synthesis of Py_{20}-PDI_{19}-PEG_{10} Copolymer

The synthesis method is in agreement with that of the TPA_{21}-PDI_{19}-PEG_{10} polymer, and the feeding dosages are as follows: G3 catalyst (8.3 mg, 0.0114 mmol), Py monomer (84.6 mg, 0.229 mmol), PDI monomer (200 mg, 0.229 mmol), and PEG monomer (92.4 mg, 0.114 mmol). At the end of the reaction, Py_{20}-PDI_{19}-PEG_{10} polymer (252.1 mg, 67%) was obtained.

Synthesis of Cz_{19}-PDI_{18}-PEG_{10} Copolymer

The synthesis method is in agreement with that of the TPA_{21}-PDI_{19}-PEG_{10} polymer, and the feeding dosages are as follows: G3 catalyst (8.3 mg, 0.0114 mmol), Cz monomer (107 mg, 0.2285 mmol), PDI monomer (200 mg, 0.229 mmol), and PEG monomer (92.4 mg, 0.114 mmol). At the end of the reaction, Cz_{19}-PDI_{18}-PEG_{10} polymer (239.6 mg, 60%) was obtained.

Preparation of micelles

The micelles are prepared by solvent-induced self-assembly. 10 mg of the copolymer is homogeneously dissolved into a mixture of THF (2 mL). 8 mL deionized water is added dropwise in the process of 10 × 200 μL, 10 × 300 μL, 10 × 300 μL, and the addition interval is

10 s. Subsequently, the solution is frozen and freeze dried to get the pure micelle for further experiments.

Characterization

^{1}H NMR is recorded on a INOVA 400MHz FT-NMR spectrometer at 25 °C. GPC is measured on a Waters1515 series using THF as the eluent with a flow rate of 1.0 mL/min, and calibrated with monodisperse polystyrene standards. The optical properties of the materials are characterized by Shimadzu UV-3600. The morphology of the samples is observed by SEM (Hitachi SU8230), and TEM (Hitachi HT7700). The elemental mapping is performed by HRTEM (FEI TecnaiG2F20). DLS is carried out by Marvern Zetasizer Nano zs. Steady/transient-state PL spectra are characterized by Edinburgh FLS920 and Edinburgh FLS980 spectrophotometer. The concentration of BPA is analyzed by high performance liquid chromatography (HPLC, Ultimate 1000). Reactive oxygen species are detected by ESR (Bruker A300 EPR). LC–MS (Waters ZQ2000) is used to detect and analyze the intermediate products.

Electrochemical experiments

Electrochemical experiments are conducted on CHI660E (chenhua Shanghai) by a standard three-electrode system. Ag/AgCl and a platinum wire is used as the reference and counter electrode respectively. Specially, the working electrode is prepared as follows, 5 mg micelle, 100 μL Nafion solution, 200 μL pure water and 700 μL acetonitrile are mixed and sonicated for 1 h to obtain a homogeneous mixture. Then 20 μL is dropped on the GCE and dried under the infrared light. For the transient photo response, electrochemical impedance spectroscopy (EIS) and Mott–Schottky measurements, the working electrode is prepared by dropping 60 μL of the micelle mixture on an indium tin oxide glass (2 cm × 2 cm). The measurement is performed in a Na_2SO_4 (0.1 M) aqueous solution and the light source is a 300 W xenon lamp. The E_{VB} values are calculated based on the following equation:

$$E_{VB}=E_g+E_{CB}$$

Quantitative analysis of ROS

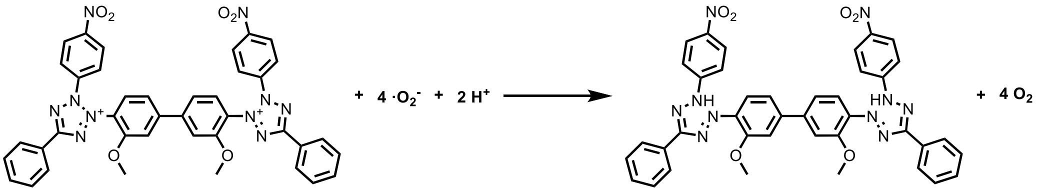

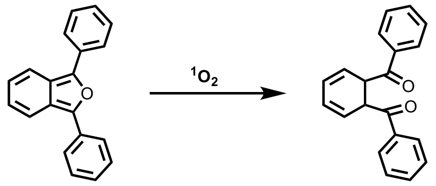

Quantitative analysis of $\bullet O_2^-$. Nitrotetrazolium blue chloride (NBT) is used as a probe molecule to detect $\bullet O_2^-$. A 300 W Xe lamp (λ > 420 nm) is used as the light source during the measurement. The concentration of $\bullet O_2^-$ is determined by UV-vis spectrophotometer. 5 mg of photocatalyst is dispersed in 24 mL deionized water, and the suspension is well dispersed by ultrasonication. Then 1 mL of 1 mM NBT aqueous solution is added to the suspension. 2.5 mL sample is removed at a specific time, filtered through a 0.22 μm filter to remove the photocatalysts. The photocatalytic generation of $\bullet O_2^-$ is determined by the degradation of NBT, which is detected by the absorbance change at the wavelength of 259 nm. The mole ratio of generated $\bullet O_2^-$ and reacted NBT was 4:1[3].

Quantitative analysis of $^1O_2$. $^1O_2$ is detected by determining the amount of decomposition of 1,3-diphenylisobenzofuran (DPBF). A 300 W Xe lamp with a 550 nm cutoff filter is used as the light source during the measurement. The concentration of $^1O_2$ is determined by UV-vis spectrophotometer (absorption maximum at 411 nm). $^1O_2$ is chemically allowed to react with DPBF at a molar ratio of 1:1[4].

To obtain the calibration curve, NBT/DPBF solutions with known concentrations are pre-measured by UV-vis spectrometer (**Figure S7c and S8h**). Based on the linear relationship between the signal intensity and NBT/DPBF concentration, the $\bullet O_2^-$/$^1O_2$ concentrations of the samples can be obtained[5].

Quantum yields of $^1O_2$

Singlet oxygen quantum yield (Φ_Δ) is measured according to the reported procedure[4, 6]. The Φ_Δ of micelles are obtained based on the Rose bengal (RB) reference in MeCN (Φ_Δ = 0.54)[4]. After obtaining the nearly same UV absorption intensity at 550 nm by separately adjusting the concentrations of Py_{20}-PDI_{19}-PEG_{10}, Cz_{19}-PDI_{18}-PEG_{10}, and TPA_{21}-PDI_{19}-PEG_{10} and RB in MeCN (**Figure S8b**), the same amount of DPBF is added to the above solutions. The absorbance is recorded at every 1 min. The absorbance maxima of DPBF at 411 nm are then plotted versus time and the slope is determined for each photosensitizer. Φ_Δ is calculated using the following formula[6]:

$$\frac{\Phi_\Delta(\text{photocatalyst})}{\Phi_\Delta(\text{RB})} = \frac{\text{slope(photocatalyst)}}{\text{slope(RB)}}$$

Adsorption experiments

BPA is selected as the target pollutant to evaluate the adsorption and photocatalytic properties of micelle. In the adsorption experiment, 10 mg micelle is added to 20 mL of deionized water. The stock solution of the adsorbent is sonicated and dispersed for 5 min before performing the adsorption experiment. Subsequently, 5 mL high concentration of BPA solution (250 ppm) is added to obtain the target BPA solution with an initial concentration of 50 ppm ($C_{catalyst}$ = 0.4 g L^{-1}). The mixed solution is stirred in a thermostatic water bath at 400 rpm and taken up for a fixed time interval (10 s, 30 s, 60 s, 90 s, 120 s, 180 s, 240 s, 300 s), then the extracted solution each time is filtered through a membrane with a pore size of 0.22 μm and prepared for HPLC analysis with the mobile phase (V_{MeOH} : V_{H2O} = 70:30) flow rate of 1 mL/min and the elution time of 7 min.

Photocatalytic activity measurements

The photocatalytic experiment is started after reaching the adsorption equilibrium. To avoid thermal catalysis due to high temperature, temperature was maintained at 25 °C by using circulating condensate water. Under 300 W Xe lamp (λ > 420 nm, 300 mW cm^{-2}) irradiation, 0.6 mL of the sample is taken up every 5 min, then filtered through a membrane with a pore size of 0.22 μm and analyzed by HPLC, so the BPA concentration in the solution (outside the micelle) is obtained. In the free radical trapping experiments, p-BQ, IPA, β-carotene and EDTA-2Na are individually used as scavengers of superoxide radicals ($\bullet O_2^-$), hydroxyl radicals (•OH), singlet oxygen ($^1O_2$), and holes (h^+). For BPA desorption experiments, MeOH is selected as the solvent to allow the desorption of BPA from the micelle. During the photocatalytic process, 0.6 mL of the samples are withdrawn at a fixed time interval. The sample solution in the vial is dried at 70 °C. Subsequently, the same volume of MeOH (0.6 mL) is added to the vial, which then sonicated for 2 h to enable full desorption of BPA. The micelle in MeOH is filtered through an organic membrane with a pore size of 0.22 μm and the filtrate is prepared for HPLC analysis, thus the BPA concentration in the whole system (both inside and outside the micelle) can be obtained. For the cyclic adsorption and photodegradation experiments, the micelle is re-collected by means of freeze-drying before the next the photocatalytic performance experiment.

Computational methods

Initially, ground-state geometry optimizations of the systems are performed. Considering the large molecular size, the hybrid B3LYP functional within density functional theory (DFT)[7],

combined with the 6-31G(d) basis set[8], is employed to strike a balance between computational efficiency and accuracy. This basis set includes polarization functions on heavy atoms to enhance the description of their electron density[8b, 9]. On this basis, time-dependent density functional theory (TDDFT) is used to calculate the excited-state properties of the molecules. To achieve a more accurate description of the excited states, the long-range corrected functional CAM-B3LYP is selected[10]. This is combined with the 6-311+G(d,p) basis set[11], which incorporates both diffuse and polarization functions. The inclusion of diffuse functions is crucial for accurately describing the diffuse distribution of excited-state electrons in regions far from the nucleus.

Supplementary Figures

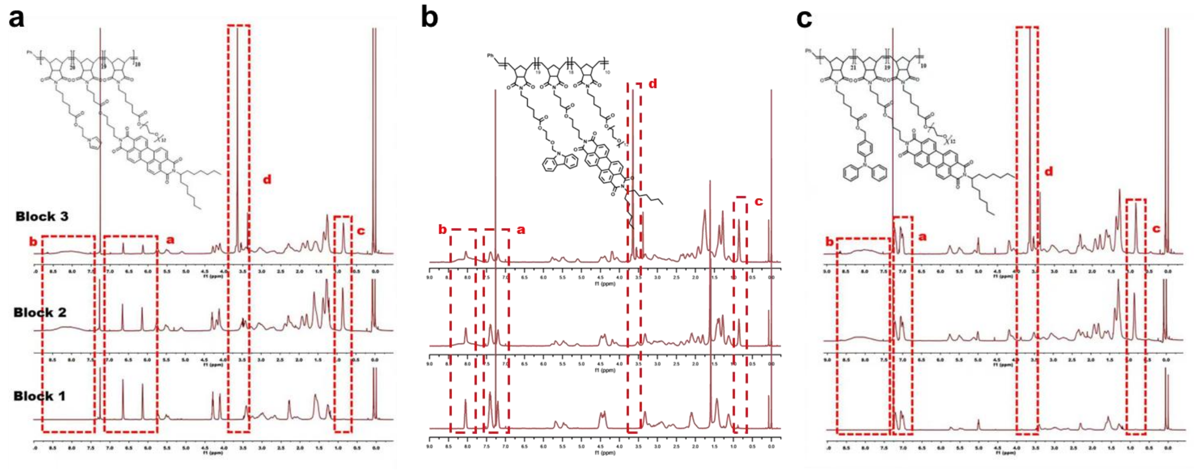

Figure S1. ^{1}H NMR spectra of block1, block2, block3 in the synthesis of (a) the Py_{20}-PDI_{19}-PEG_{10}, (b) the Cz_{19}-PDI_{18}-PEG_{10} polymer and (c) the TPA_{21}-PDI_{19}-PEG_{10} polymer.

Note: As is shown in **Figure S1a**, the peaks centered from 6.00 - 6.80 ppm (a) are classified into the characteristic peaks of Py monomer, potently verifying the successful polymerization. Similarly, the peaks at 7.14-7.50 ppm (a) (**Figure S1b**) which are attributed to Cz monomer and the peaks at 6.7-7.3 ppm (a) (**Figure S1c**) which correspond to TPA monomer, respectively certify the successful polymerization of both Cz and TPA monomer. In addition, the appearance of the characteristic peaks of PDI at 7.5-8.5 ppm (b) and 0.18-0.89 ppm (c), and of PEG at 3.61-3.67 ppm (d) is all observed in the ^{1}H NMR spectra of the three polymers (**Figure S1**), which indicates the polymerization between PDI and PEG monomer, further elucidating the successful synthesis of polymers.

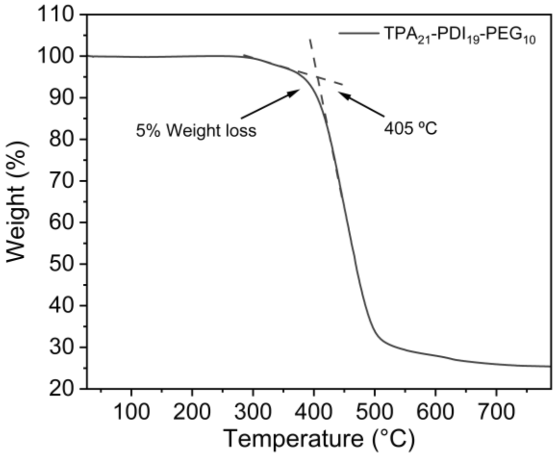

Figure S2. Thermogravimetric curve of the TPA_{21}-PDI_{19}-PEG_{10} polymer.

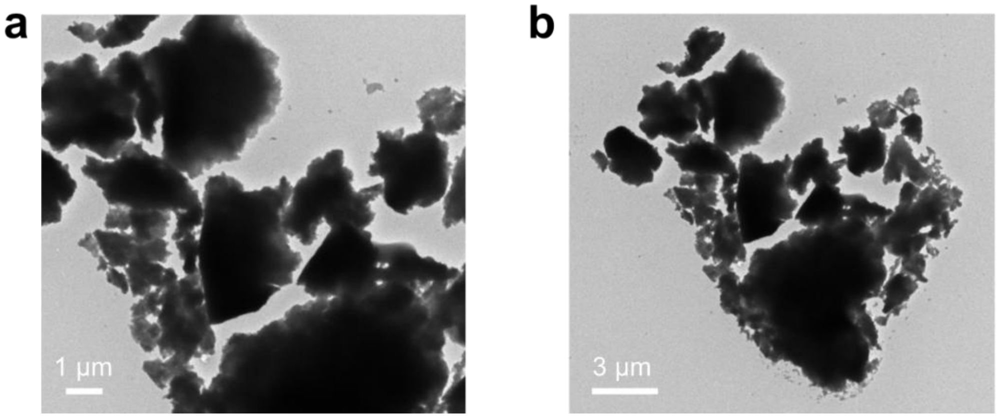

Figure S3. TEM images of the TPA_{21}-PDI_{19}-PEG_{10} polymer.

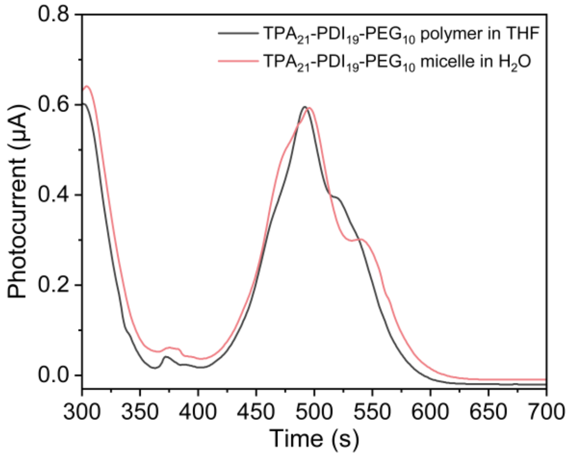

Figure S4. UV-vis absorption spectra of the TPA_{21}-PDI_{19}-PEG_{10} polymer before and after self-assembly.

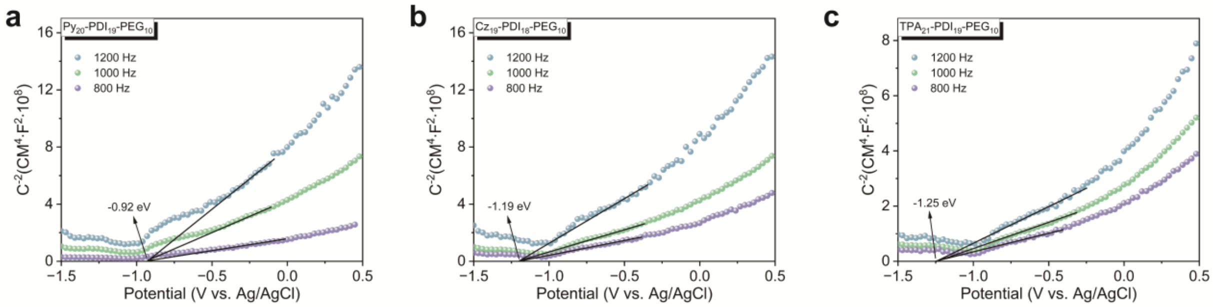

Figure S5. Mott-Schottky plots of (a) Py_{20}-PDI_{19}-PEG_{10}, (b) Cz_{19}-PDI_{18}-PEG_{10}, and (c) TPA_{21}-PDI_{19}-PEG_{10} micelles at different frequencies.

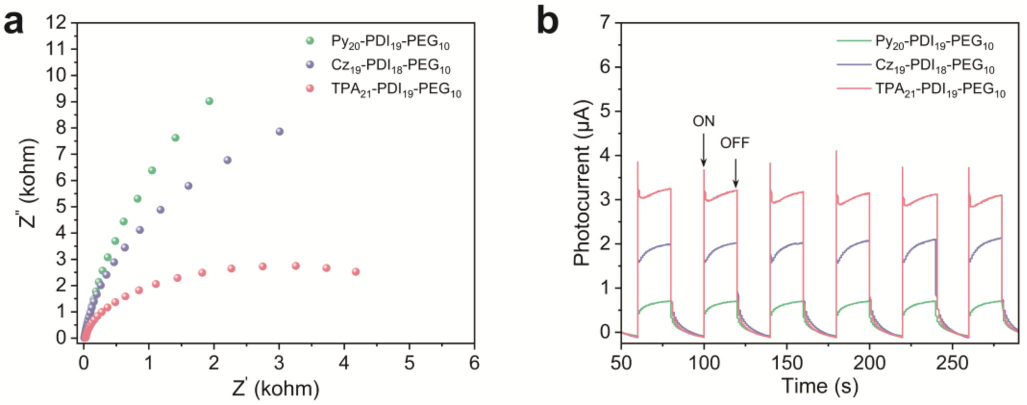

Figure S6. (a) Electrochemical impedance, and (b) photocurrent responses of different micelle samples.

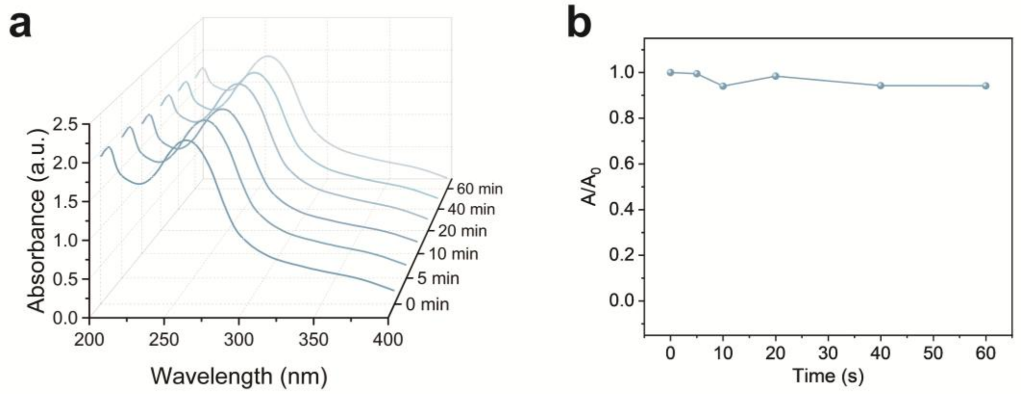

Figure S7. (a) Time-dependent UV/vis absorption spectra of NBT in deionized water upon visible light irradiation ($\lambda > 420$ nm) without catalyst. (b) The NBT degradation profile under light irradiation ($\lambda > 420$ nm).

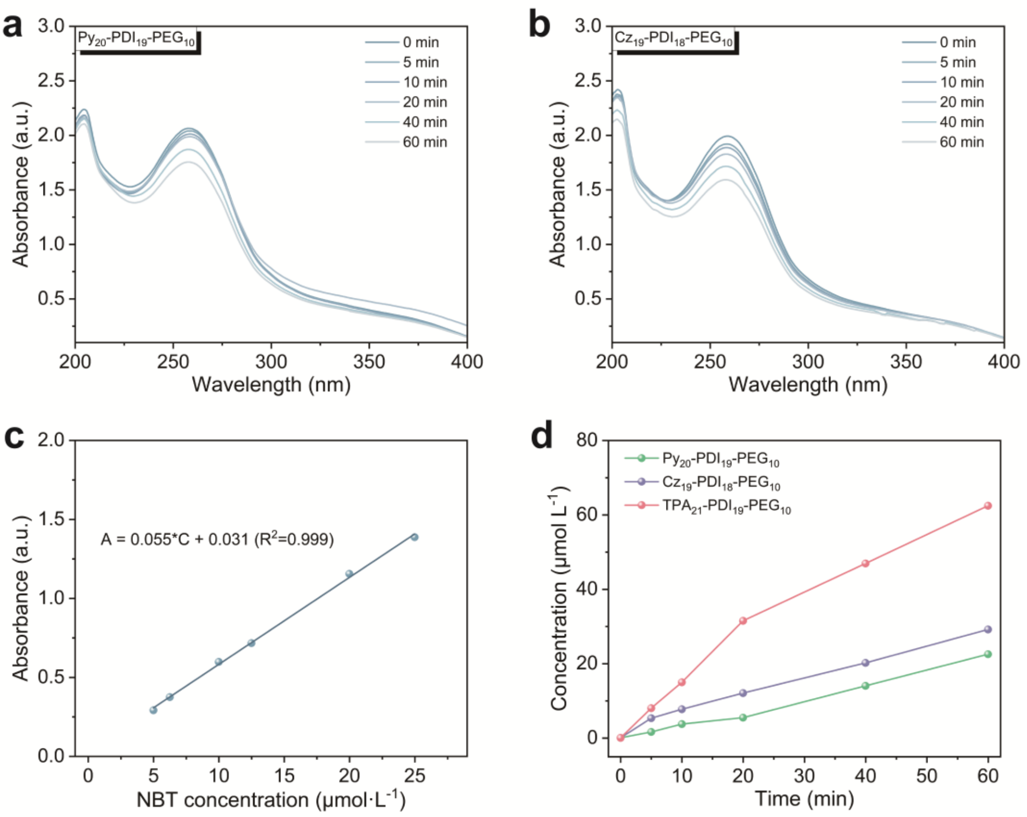

Figure S8. Time-dependent UV/vis absorption spectra of NBT in deionized water upon visible light irradiation ($\lambda > 420$ nm) in the presence of (a) Py_{20}-PDI_{19}-PEG_{10} micelle, and (b) Cz_{19}-PDI_{18}-PEG_{10} micelle. (c) The calibration curve of NBT at the absorption peak of 259 nm. (d) The concentration of $\bullet O_2^-$ generated by different micelle samples.

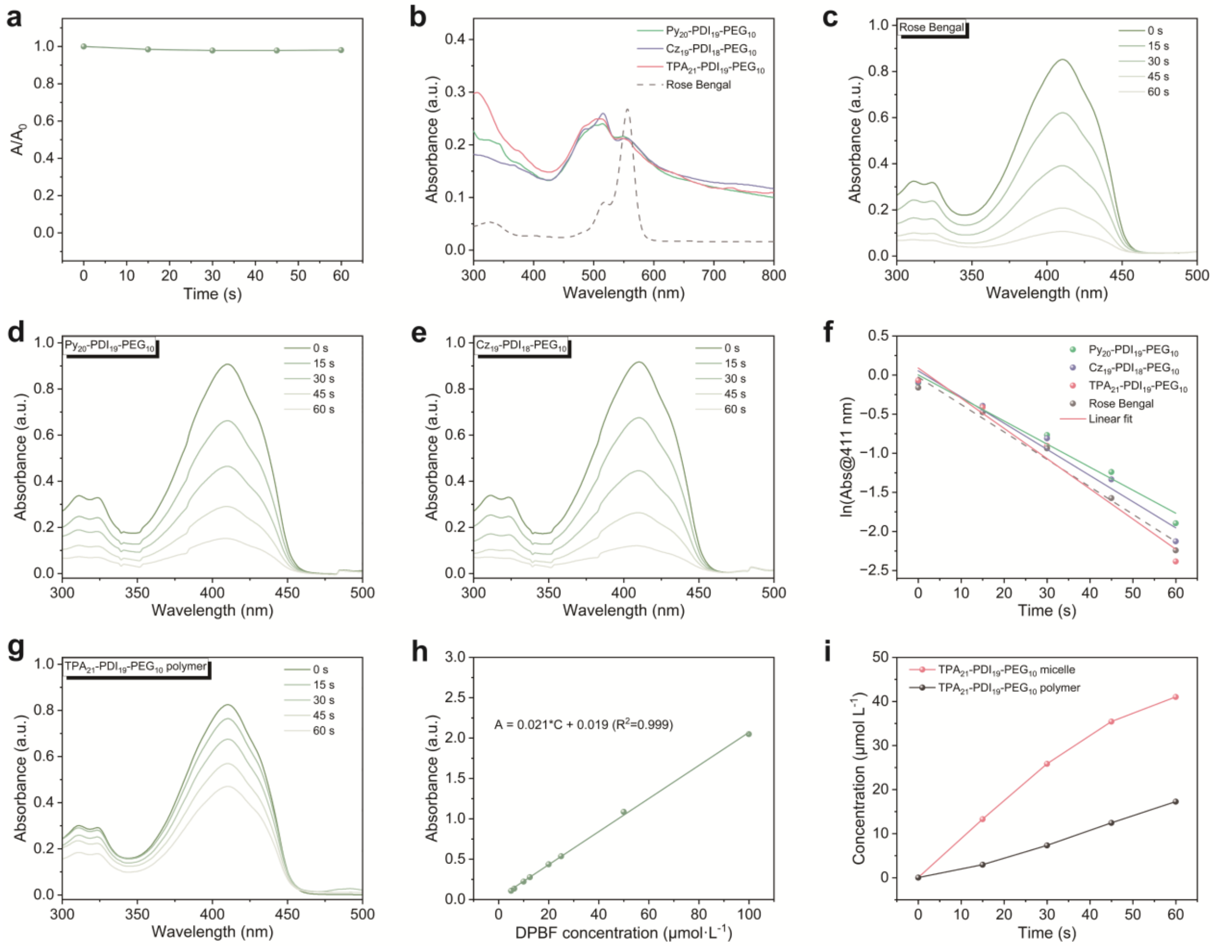

Figure S9. (a) The DPBF degradation profile under light irradiation at a wavelength of 550 nm. (b) UV/vis spectra of different micelles and RB in MeCN. Time-dependent UV/vis absorption spectra of DPBF in MeCN upon light irradiation (λ = 550 nm) in the presence of (c) Rose Bengal, (d) Py_{20}-PDI_{19}-PEG_{10} micelle, and (e) Cz_{19}-PDI_{18}-PEG_{10} micelle. (f) The logarithmic value of maximum absorbance of DPBF at 411 nm plotted versus time. (g) Time-dependent UV/vis absorption spectra of DPBF in MeCN upon visible light irradiation (λ = 550 nm) in the presence of TPA_{21}-PDI_{19}-PEG_{10} polymer. (h) The calibration curve of DPBF at the absorption peak of 411 nm. (d) The concentration of $^1O_2$ generated by TPA_{21}-PDI_{19}-PEG_{10} before and after self-assembly.

Note: When the incident light wavelength is equal to 550 nm, the self-sensitized degradation of DPBF is almost unobservable. Therefore, to minimize interference from non-specific degradation, the wavelength at 550 nm is selected as the irradiation wavelength for DPBF degradation.

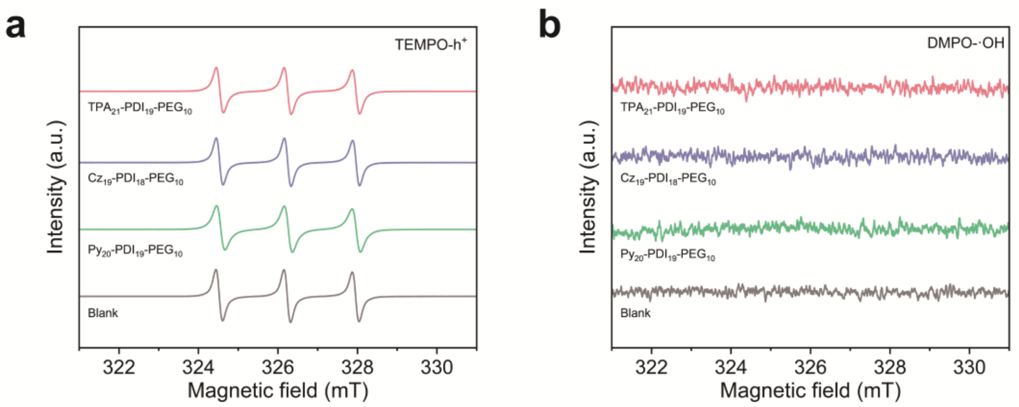

Figure S10. ESR signal of (a) TEMPO-h^+ and (b) DMPO-•OH in different micelle samples upon light irradiation.

Note: Other possible active species that might be produced in photocatalysis process are identified by ESR analysis. As can be seen in **Figure S9a**, under visible light illumination, the diminishment of the signal of TEMPO-h^+ denotes the production of h^+. However, the signal of DMPO-•OH is failed to be detected after light irradiation (**Figure S9b**), indicating that the samples cannot produce •OH. The HOMO energy levels of micelles are more negative than the oxidation potential of H_2O/•OH and OH^-/•OH (**Figure 3c**). This means that the holes generated by the micelles do not have sufficient energy to oxidize H_2O or OH^- into •OH.

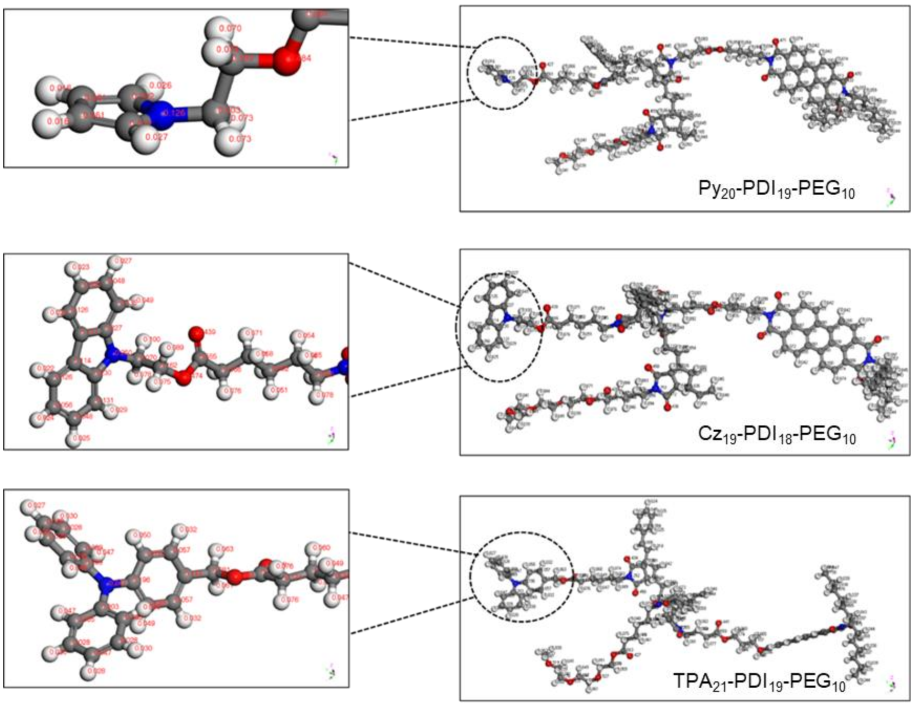

Figure S11. Mulliken atomic charges of each atom in the Py_{20}-PDI_{19}-PEG_{10}, Cz_{19}-PDI_{18}-PEG_{10}, and TPA_{21}-PDI_{19}-PEG_{10} polymers.

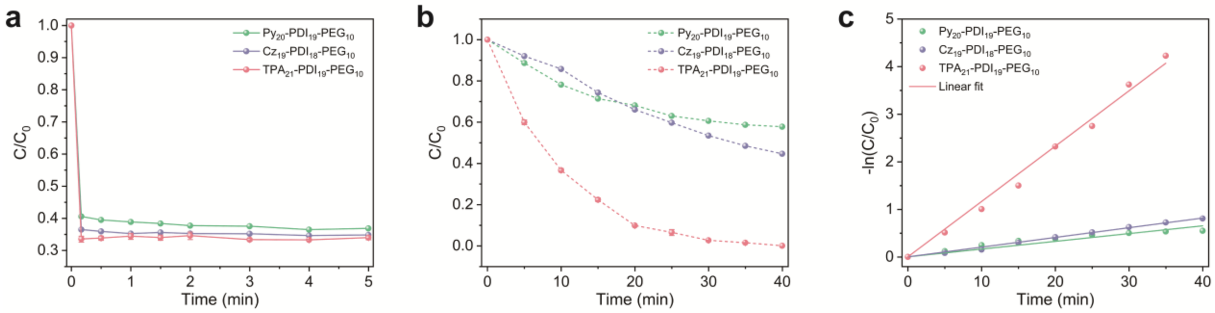

Figure S12. (a) Adsorption curves of BPA over different micelles. (b) Degradation curves of BPA in the whole system. (d) Fitting curves of the pseudo-first-order kinetic model.

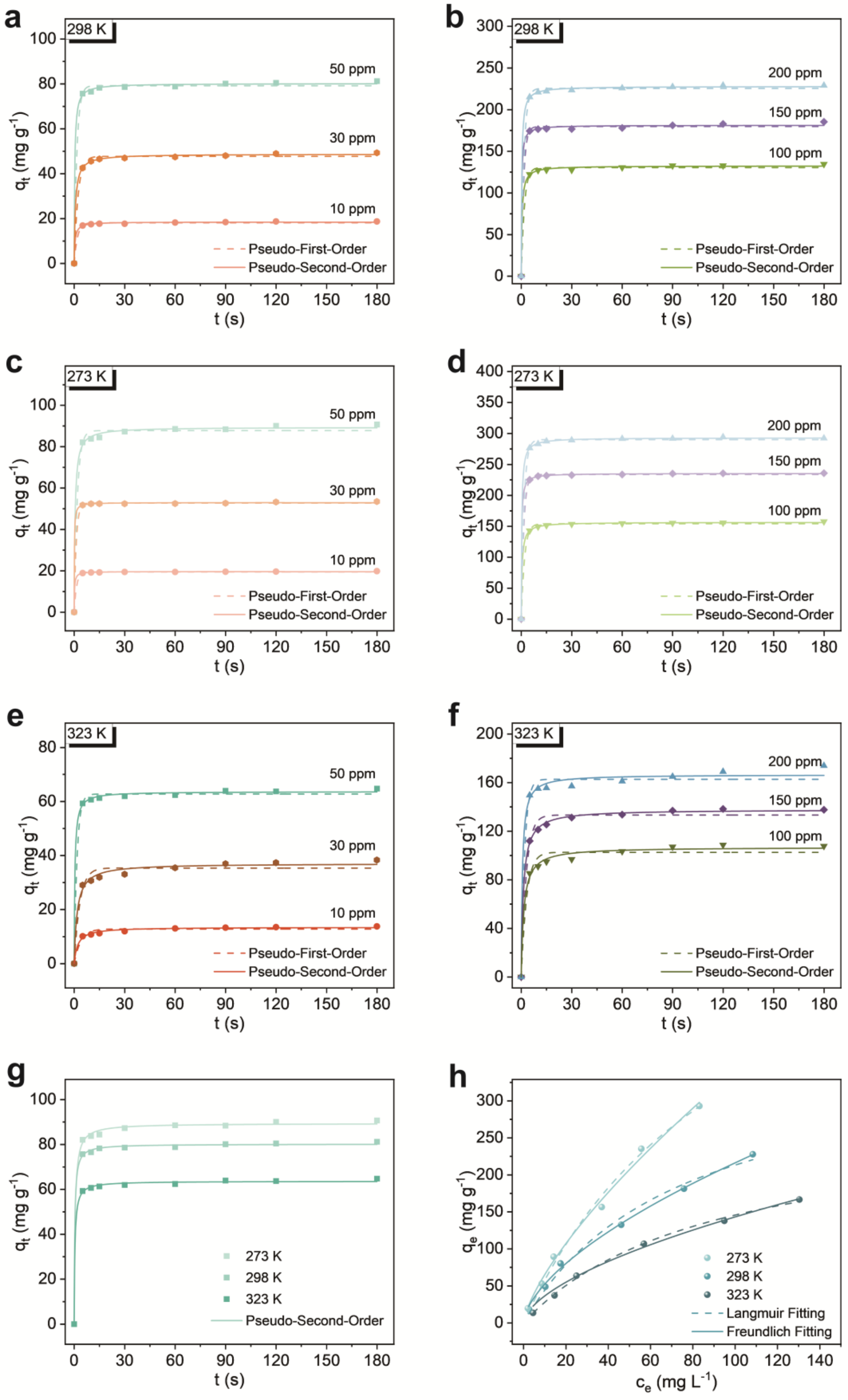

Figure S13. (a) Adsorption kinetics and the corresponding non-linear fit curves of the TPA_{21}-PDI_{19}-PEG_{10} micelle to BPA of different concentrations at (a-b) 298 K, (c-d) 273 K, and (e-f) 323 K. (g) Adsorption kinetics and the corresponding non-linear fit curves of the micelle to

BPA at different temperatures. (h) Adsorption isotherms and the corresponding non-linear fit curves of the micelle to BPA at different temperatures.

Note: The pseudo-first-order (PFO, **Equation S1**) and pseudo-second order (PSO, **Equation S2**) kinetic models are applied to mathematically evaluate the adsorption kinetics of the TPA_{21}-PDI_{19}-PEG_{10} micelle to BPA (**Figure S13a-f**). The parameters in the two models (k_1, q_e and k_2) are determined from non-linearized fit plots of q_t versus t (**Table S8**). It is evident that the correlation coefficients (R^2) in the PSO model are all higher than those in the PFO model. Meanwhile, The calculated adsorptioncapacities from PSO model are in more agreement with the experimental results. Therefore, it can be concluded that BPA adsorption on the micelle is better described by the PSO kinetic model than the PFO kinetic model. Additionally, the adsorption kinetics of the micelle toward BPA are directly compared at different temperatures (**Figure S13g**), and the relevant parameters are summarized in **Table S9**. According to the Arrhenius equation (**Equation S3**), the calculated activation energy for BPA adsorption onto the micelle is approximately 8.60 kJ mol^{-1}, indicating that the adsorption mechanism is dominated by physical adsorption[12].

$$q_t = q_e\left(1 - e^{-k_1 t}\right) \qquad \textbf{(S1)}$$

$$q_t = \frac{k_2 q_e^2 t}{1 + k_2 q_e t} \qquad \textbf{(S2)}$$

Here, q_t (mg g^{-1}) and q_e (mg g^{-1}) represent the amounts of BPA adsorbed per unit mass of TPA_{21}-PDI_{19}-PEG_{10} micelle at time t (s) and at equilibrium, respectively. k_1 (s^{-1}) is the adsorption rate constant for the PFO, while k_2 (g mg^{-1} s^{-1}) denotes the adsorption rate constant for the PSO.

$$\ln k_2 = \ln A - \frac{E_a}{RT} \qquad \textbf{(S3)}$$

Here, E_a (kJ mol^{-1}) is defined as the activation energy of the adsorption process, k_2 (g mg^{-1} s^{-1}) is the adsorption rate constant under PSO kinetics, R (8.314 J mol^{-1} K^{-1}) is the gas constant, A is the Arrhenius frequency factor, and T (K) represents the absolute temperature of the adsorption process.

Furthermore, the Langmuir (**Equation S4**) and Freundlich models (**Equation S5**) are used to fit the adsorption isotherms of BPA adsorbed on the TPA_{21}-PDI_{19}-PEG_{10} micelle (**Figure S13h**). From the fitting results (**Table S10**), the calculated R^2 values demonstrate that the Freundlich model is more appropriate than the Langmuir model for fitting the adsorption isotherms of the micelle, indicating that adsorption of the micelle to BPA is multilayer adsorption[13]. What's more, a thermodynamic study is performed based on the Gibbs equations (**Equation S6-7**), and

the corresponding parameters are displayed in **Table S10**. The negative values of ΔG and ΔH indicate that the adsorption process is spontaneous and exothermic. Hence, the decrease in temperature is conducive to the enhancement of adsorption capacity. Concurrently, ΔS < 0 suggests that the adsorption process reduces the randomness of BPA molecules in the solution[14].

$$q_e=\frac{q_m k_L c_e}{1+k_L c_e} \quad \text{(S4)}$$

$$q_e=k_F c_e^{1/n} \quad \text{(S5)}$$

In these equations, c_e (mg L^{-1}) represents the equilibrium concentration of BPA, q_e (mg g^{-1}) represents the equilibrium adsorption capacity of BPA, c_0 (mg L^{-1}) represents the initial concentration of BPA, q_m (mg g^{-1}) represents the maximum adsorption capacity of BPA, k_L (L mg^{-1}) represents the Langmuir model constant, n represents the favorable factor for adsorption, k_F (mg g^{-1}) represents the Freundlich model constant.

$$\ln\frac{q_e}{c_e}=\frac{\Delta S}{R}-\frac{\Delta H}{RT} \quad \text{(S6)}$$

$$\Delta G=\Delta H-T\Delta S \quad \text{(S7)}$$

Within this scope, ΔH, ΔS, and ΔG represent the standard enthalpy (kJ mol^{-1}), entropy (kJ mol^{-1} K^{-1}), and Gibbs free energy (kJ mol^{-1}) of the adsorption process.

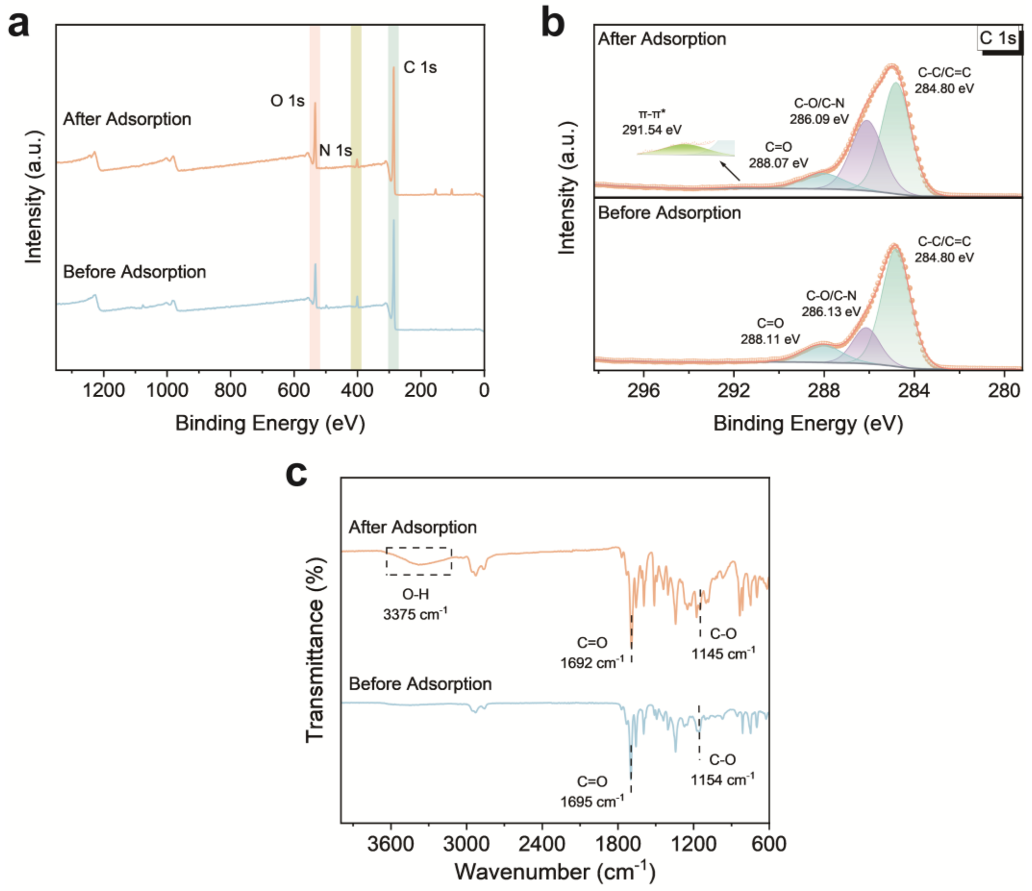

Figure S14. (a) Survey XPS spectra, (b)high-resolution C 1s spectra, and (c) FTIR spectra of the TPA_{21}-PDI_{19}-PEG_{10} micelle before and after BPA adsorption.

Note: Given the structural characteristics of the TPA_{21}-PDI_{19}-PEG_{10} micelle and BPA molecules, it can be inferred that hydrogen bonding, π-π interactions, and hydrophobic interactions are involved in the adsorption process. The adsorption mechanism is further elucidated by comparing the XPS and FT-IR spectra of the micelle before and after BPA adsorption. As shown in the survey XPS spectra (**Figure S14a**), the intensities of C 1s and O 1s peaks increased markedly after BPA adsorption, indicating the successful attachment of BPA molecules, which are rich in carbon- and oxygen-containing functional groups[15]. High-resolution C 1 s spectra reveal systematic binding energy shifts in characteristic carbon bonds after BPA adsorption (**Figure S14b**). The binding energy peaks associated with C=O and C-N/C-O groups shift to lower positions, and a small stacked feature peak appears at 291.5 eV, confirming the π-π interactions between the adsorbent and BPA[16]. Moreover, after BPA adsorption, the new peak at 3375 cm^{-1}, which corresponds to the streching vibration of O-H in BPA, can be clearly observed in the FT-IR spectra of TPA_{21}-PDI_{19}-PEG_{10} micelle (**Figure S14c**). Meanwhile, the characteristic bands at 1145 cm^{-1} (C–O) and 1692 cm^{-1} (C=O) red-shift to 1154 and 1695 cm^{-1} respectively, consistent with the formation of hydrogen bonds[15]. In

addition, the adsorption capacity of TPA_{21}-PDI_{19}-PEG_{10} micelle for BPA increases upon the introduction of inorganic anions (**Figure S20a**), which can be attributed to a salt-induced enhancement mechanism of hydrophobic interactions[17]. These synergistic interactions discussed above collectively account for the high affinity and adsorption capacity of the TPA_{21}-PDI_{19}-PEG_{10} micelle toward BPA.

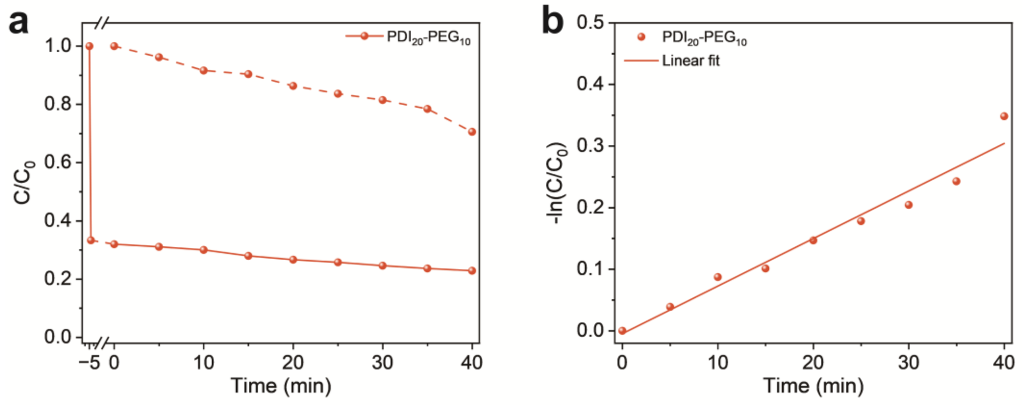

Figure S15. (a) Photodegradation curves of BPA over PDI_{20}-PEG_{10} micelle (dashed line is the BPA concentration changes in the whole system obtained by the desorption with MeOH). (b) Fitting curve of the pseudo-first-order kinetic model.

Note: Compared to three D-A type micelles, pure PDI_{20}-PEG_{10} micelle displays inferior BPA degradation performance, with a BPA removal ratio of 70.6% at 40 min. Especially, the calculated reaction rate constant is only 0.00772 min^{-1}, demonstrating a notable decrease. These experimental results underscore the critical role of the D-A structure in boosting BPA removal efficiency.

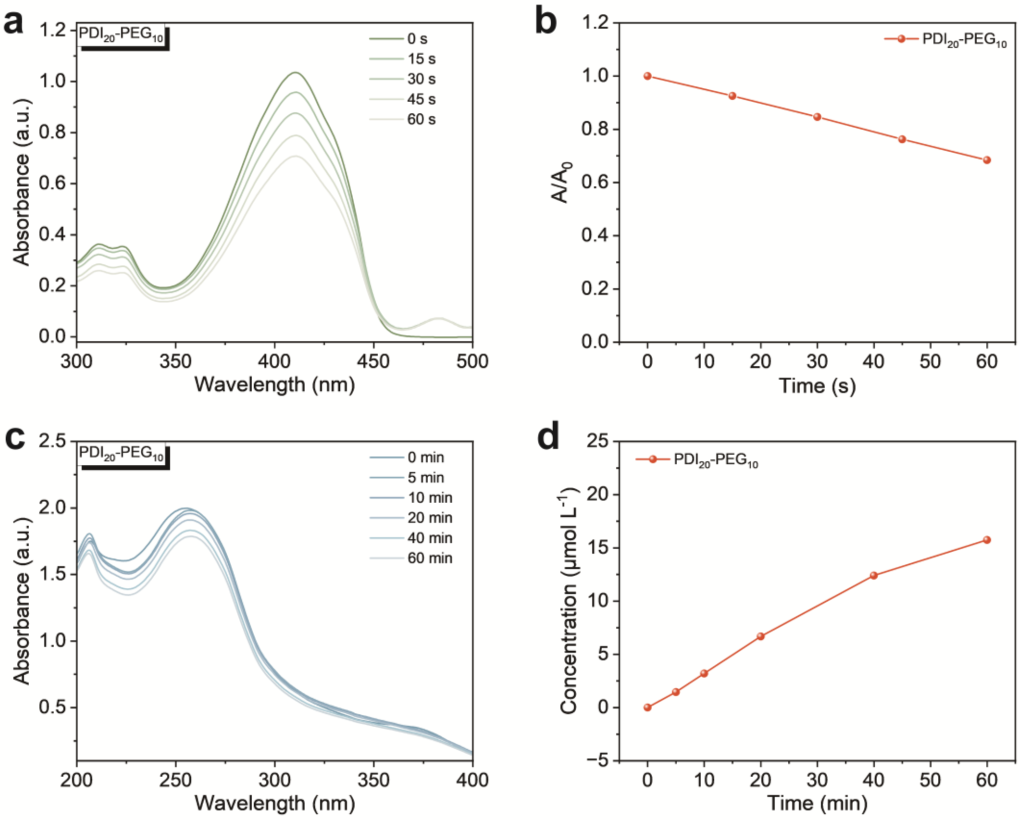

Figure S16. (a) Time-dependent UV/vis absorption spectra of DPBF in MeCN upon light irradiation (λ = 550 nm), and (c) NBT in deionized water upon visible light irradiation ($\lambda >$ 420 nm) in the presence of PDI_{20}-PEG_{10} micelle. (b) The absorbance of DPBF oxidation with PDI_{20}-PEG_{10} micelle. (d) The concentration of $^1O_2$ generated by PDI_{20}-PEG_{10} micelle.

Note: Free radical probe experiments using DPBF and NBT as probe molecules, respectively, are carried out to quantitatively compare the ROS ($^1O_2$ and $\bullet O_2^-$) generation capacity of pure PDI_{20}-PEG_{10} micelle and D-A type micelles. As is depicted in **Figure S16**, both $^1O_2$ and $\bullet O_2^-$ are generated less effectively by the PDI_{20}-PEG_{10} micelle than by the three D-A micelles, which is consistent with the previously mentioned capability of BPA removal.

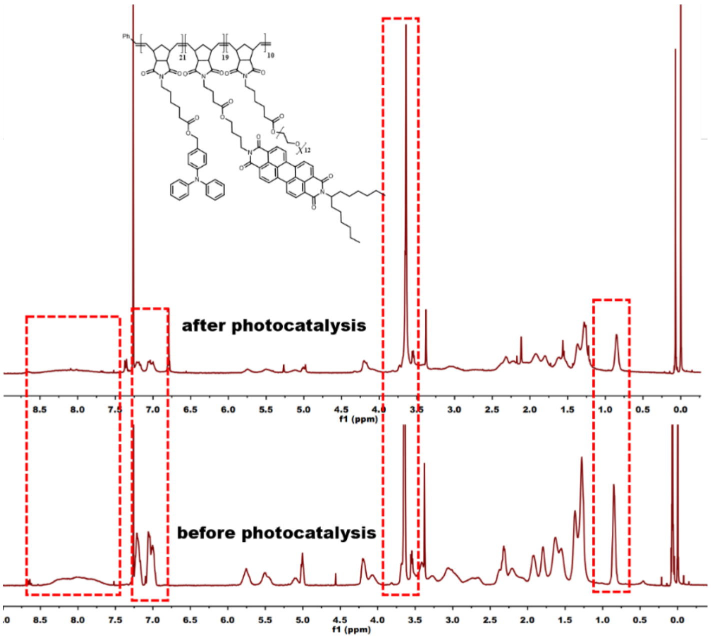

Figure S17. ^{1}H NMR spectra of the TPA_{21}-PDI_{19}-PEG_{10} polymer micelle after photocatalysis.

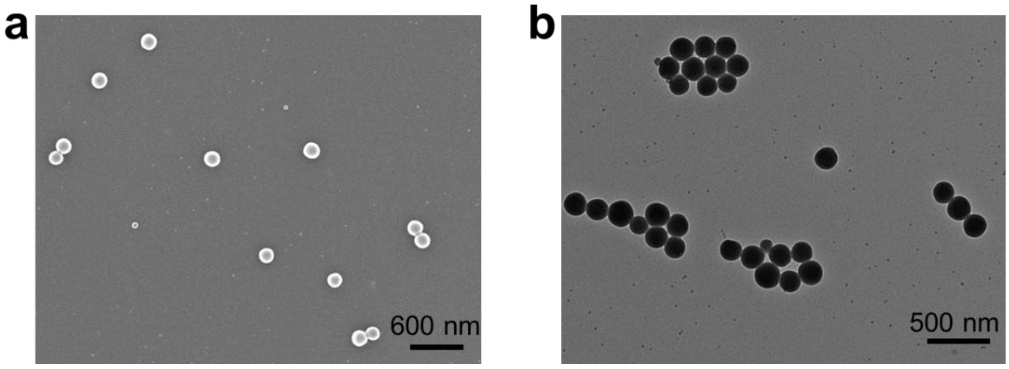

Figure S18. (a) SEM and (b) TEM images of the TPA_{21}-PDI_{19}-PEG_{10} micelle after photocatalysis.

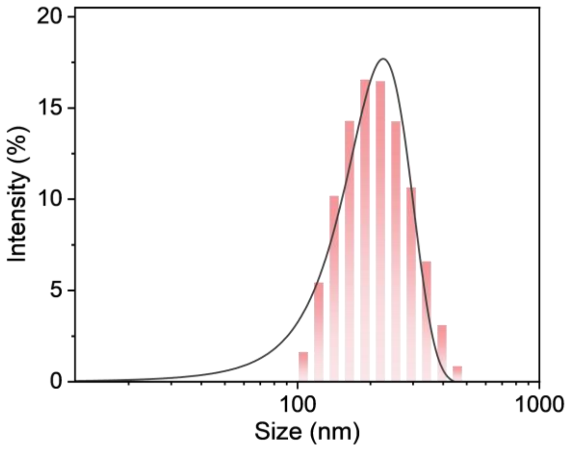

Figure S19. DLS size distribution of the TPA_{21}-PDI_{19}-PEG_{10} micelle after photocatalysis.

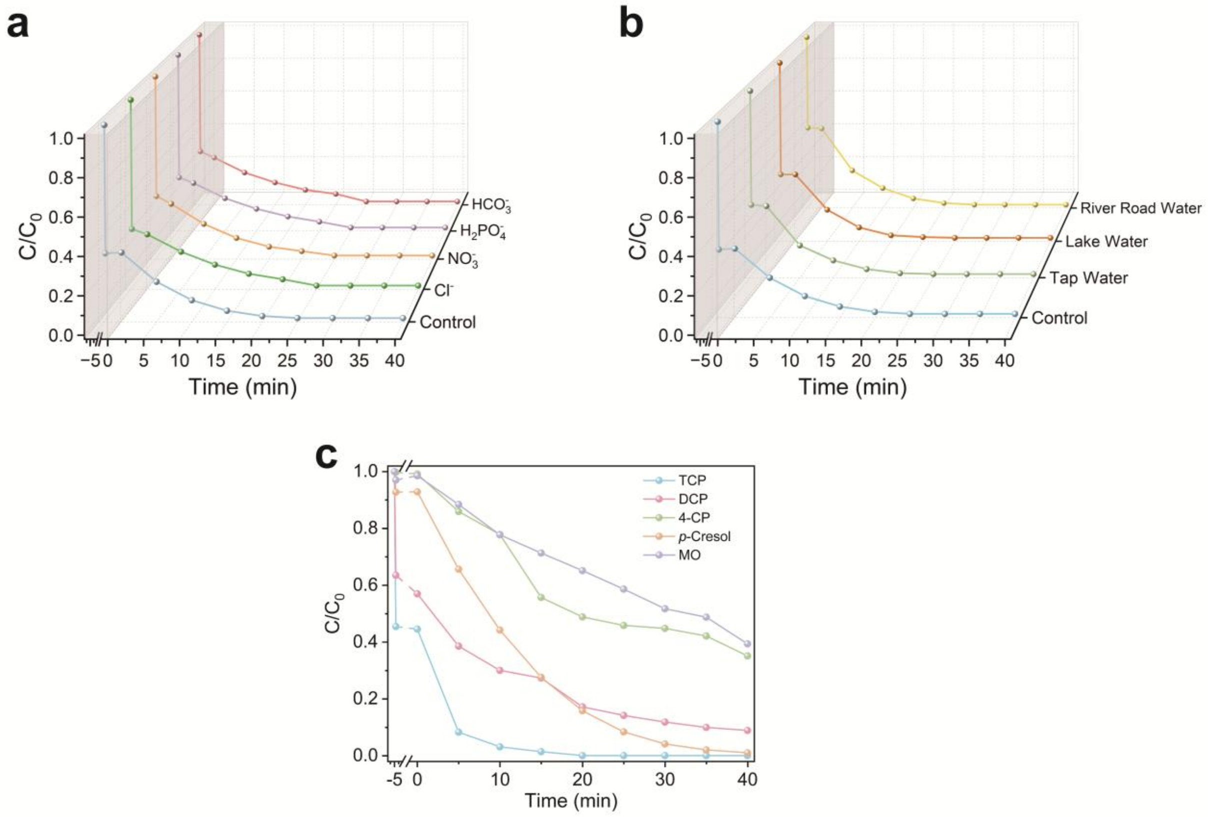

Figure S20. Adsorption and photodegradation performance of the TPA_{21}-PDI_{19}-PEG_{10} micelle to BPA (a) in the presence of different inorganic anions (100 mM) and (b) at different actual water environments. (c) Adsorption and photodegradation performance of the TPA_{21}-PDI_{19}-PEG_{10} micelle to different types of pollutants.

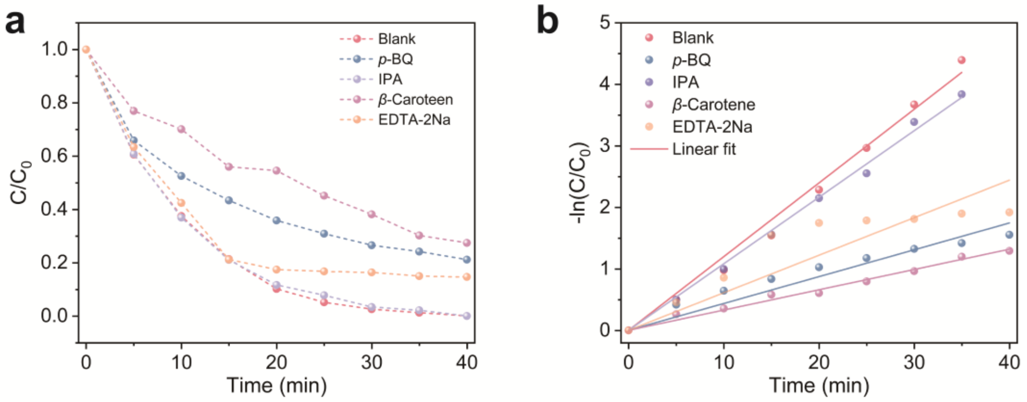

Figure S21. (a) Degradation curves of BPA in the whole system and (b) degradation rate constants of BPA by TPA_{21}-PDI_{19}-PEG_{10} micelle in the presence of different scavengers.

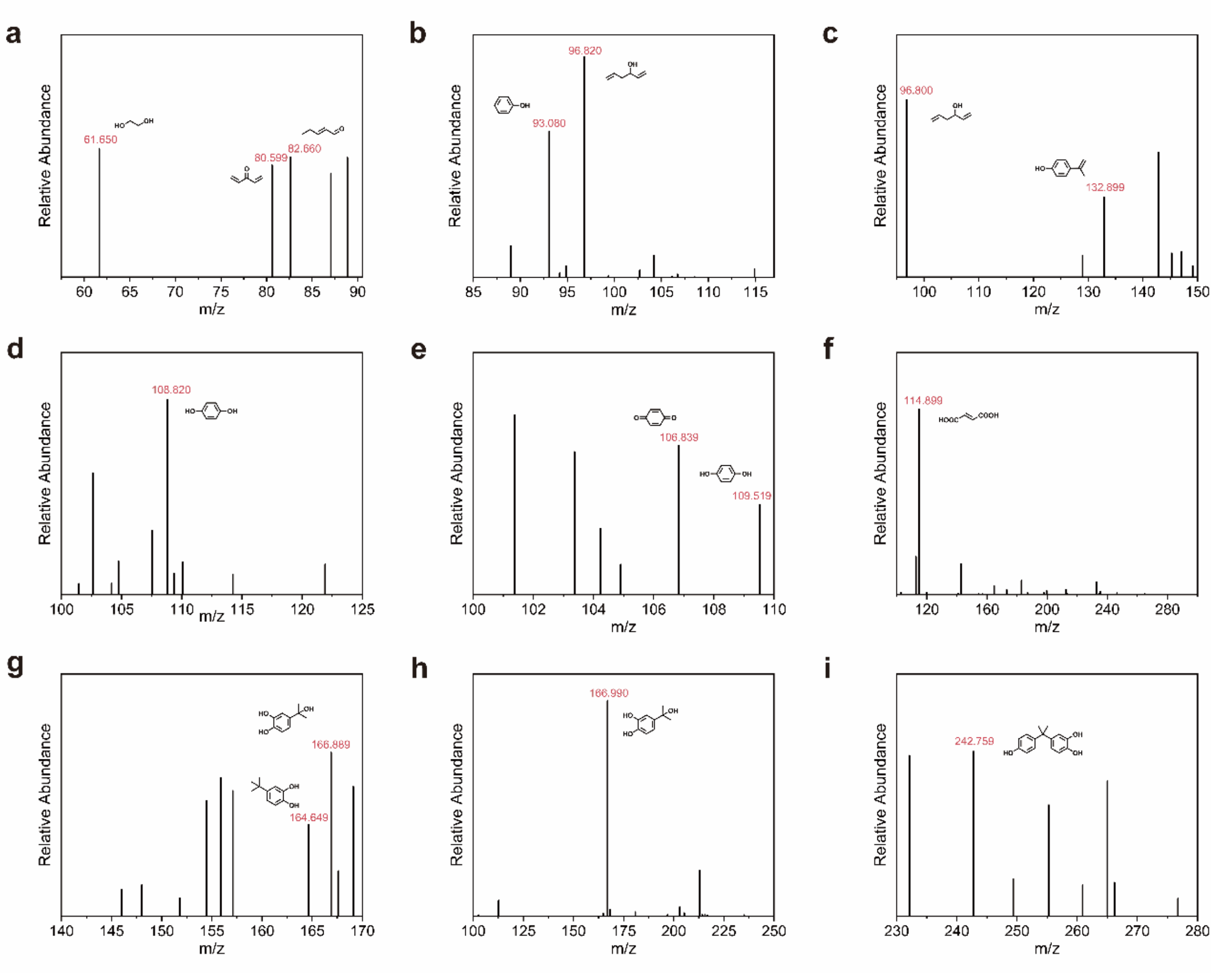

Figure S22. (a-i) LC-MS spectra of the possible intermediates during the photodegradation of BPA in the TPA_{21}-PDI_{19}-PEG_{10} micelle.

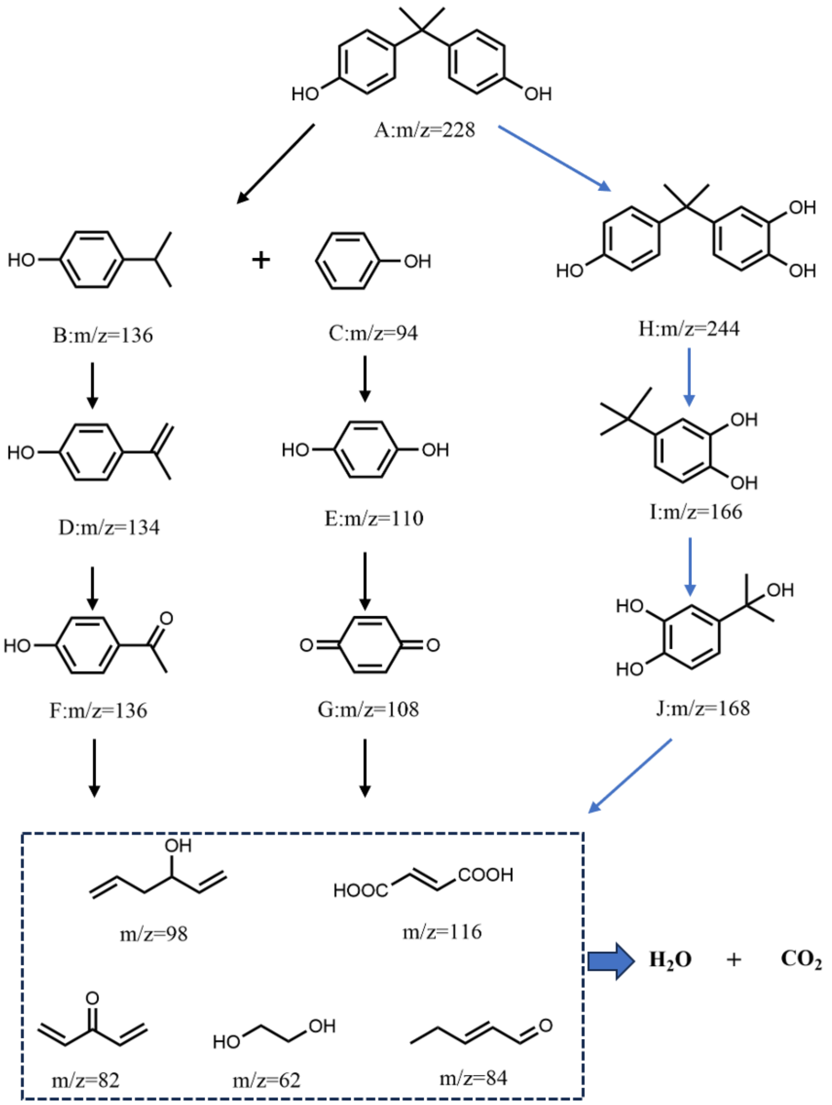

Figure S23. Possible degradation pathways of BPA in the TPA21-PDI19-PEG10 micelle.

Note: BPA can be degraded in the following two ways. In one pathway, at first, in the presence of h^+, the C-C bond is broken, and BPA is converted into 4-isopropylphenol (B: m/z=136) and phenol (C: m/z=94). Next, compound B is converted by desaturation to 4-isopropenylphenol (D: m/z=134), which is further oxidized to 1-(4-hydroxyphenyl)ethan-1-one (F: m/z=136). Whereas phenol (C) is gradually oxidized to p-phenol (E: m/z=110) and then further oxidized to p-benzoquinone (G: m/z=108). In another pathway, BPA undergoes hydroxylation to generate 4-(2-(4-hydroxyphenyl)propan-2-yl)benzene-1,2-diol (H: m/z=244), which is then transformed into 4-(tert-butyl)benzene-1,2-diol (I: m/z=166) and 4-(2-hydroxypropan-2-yl)benzene-1,2-diol (J: m/z=168)[18]. These compounds obtained in both pathways are decomposed continually, so as to produce a variety of small molecules, which is able to utterly broken down to CO_2 and H_2O in the end of the photocatalytic process.

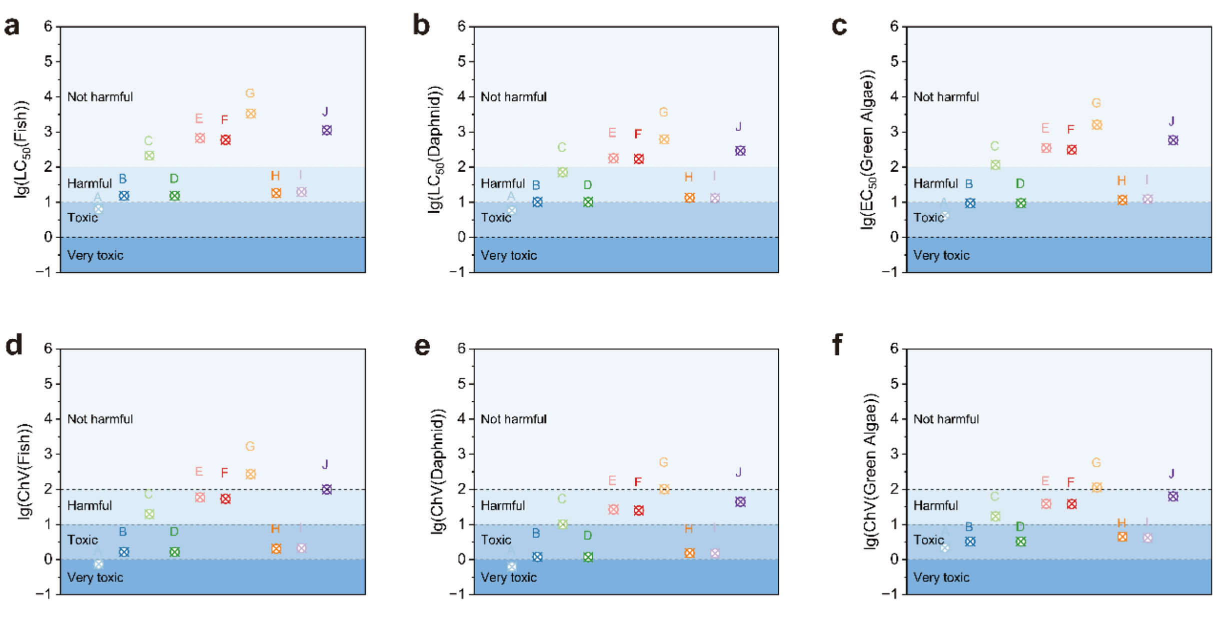

Figure S24. Predicted acute and chronic toxicities of BPA and its degradation intermediates.

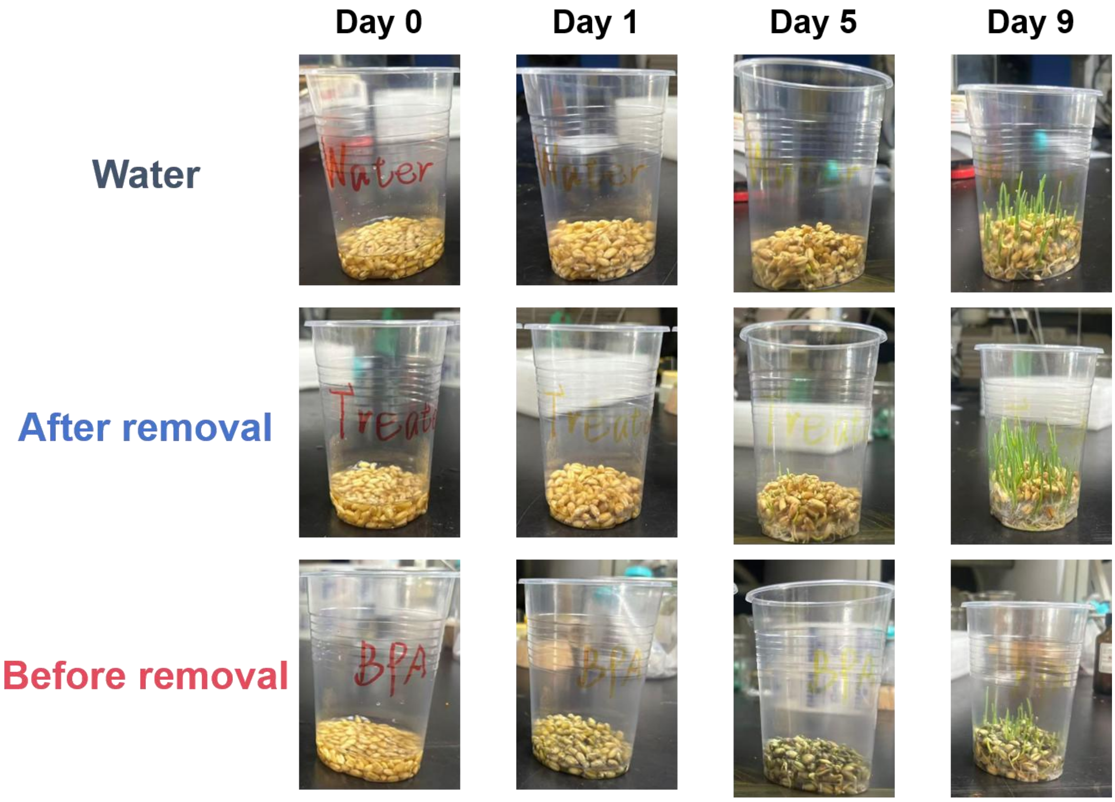

Figure S25. Photographs of wheat growth in different cultures. Water (No.1), after removal (No.2), before removal (No.3).

Notes: When the concentration of BPA exceeds 50 ppm, it not only inhibits the germination of wheat seeds, but also adversely affects the height of seedlings, ultimately leading to crop yield reduction[19]. Thus, wheat seeds are employed to intuitively assess the BPA removal efficiency of the material and the ecotoxicity of the degradation products. Through observing the growth of wheat seedlings before and after BPA removal, it is obvious to see that seeds No.3 exhibit

black discoloration and the seedling height was lower than those of the other two groups. By contrast, normal growth is restored after BPA removal by the TPA_{21}-PDI_{19}-PEG_{10} micelle, which further validates the environmental safety of the material as well as its potential for effectively eliminating BPA in aqueous solution.

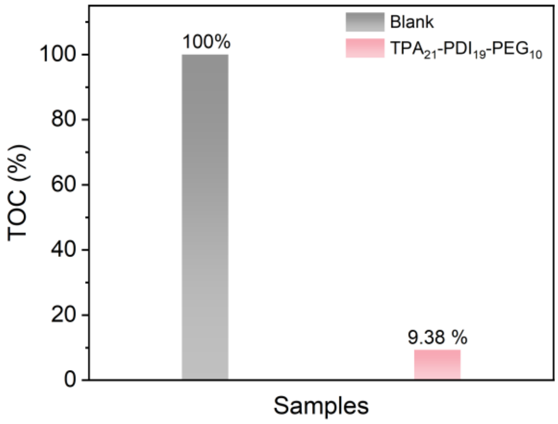

Figure S26. TOC removal of BPA by TPA_{21}-PDI_{19}-PEG_{10} micelle.

Supplementary Tables

Table S1. GPC data of block1, block2, block3 in the synthesis of different polymers.

Sample name		M_n	M_w	Poly-dispersity
Py_{20}-PDI_{19}-PEG_{10}	Block 1	7854	8993	1.145
	Block 2	25885	28888	1.116
	Block 3	34514	36435	1.056
Cz_{19}-PDI_{18}-PEG_{10}	Block 1	8313	8748	1.052
	Block 2	23307	25755	1.105
	Block 3	30881	33316	1.079
TPA_{21}-PDI_{19}-PEG_{10}	Block 1	11306	11567	1.023
	Block 2	27515	31414	1.142
	Block 3	35522	46215	1.301

Table S2. Electrochemical data (from Mott-Schottky plots) of different micelle samples.

Samples	E_{fb} (eV)	E_{CB} (eV vis. NHE)	E_{VB} (eV vis. NHE)	E_g (eV)
Py_{20}-PDI_{19}-PEG_{10}	-0.92	-0.92	1.07	1.99
Cz_{19}-PDI_{18}-PEG_{10}	-1.19	-1.19	0.82	2.10
TPA_{21}-PDI_{19}-PEG_{10}	-1.25	-1.25	0.74	1.99

Table S3. Time-resolved fluorescence decay fitting data of different micelle samples.

Samples	τ_1 (ns)/(A_1%)	τ_2 (ns)/(A_2%)	τ_{av} (ns)[a]	χ^2
Py_{20}-PDI_{19}-PEG_{10}	1.499 (57.94)	6.171 (42.06)	5.02	1.284
Cz_{19}-PDI_{18}-PEG_{10}	2.332 (40.11)	7.885 (59.89)	6.87	1.258
TPA_{21}-PDI_{19}-PEG_{10}	4.109 (25.92)	11.68 (74.08)	10.85	1.185

[a]The average fluorescence lifetime τ_{av} is calculated by the equation:

$\tau_{av}=(A_1\tau_1^2+A_2\tau_2^2)/(A_1\tau_1+A_2\tau_2)$

In this equation, A_1、A_2 respectively refers to the proportion of the fluorescence lifetime τ_1, τ_2.

Table S4. Time-resolved phosphorescence decay fitting data of different micelle samples.

Samples	τ_1 (μs)/(A_1%)	τ_2 (μs)/(A_2%)	τ_{av} (μs)[b]	χ^2
Py_{20}-PDI_{19}-PEG_{10}	1.538 (59.08)	8.539 (40.92)	7.09	2.179
Cz_{19}-PDI_{18}-PEG_{10}	1.268 (71.59)	10.195 (28.41)	8.07	4.716
TPA_{21}-PDI_{19}-PEG_{10}	1.213 (59.90)	9.456 (40.10)	9.44	1.578

[b]The average phosphorescence lifetime τ_{av} is calculated by the equation:

$\tau_{av}=(A_1\tau_1^2+A_2\tau_2^2)/(A_1\tau_1+A_2\tau_2)$

In this equation, A_1、A_2 respectively refers to the proportion of the fluorescence lifetime τ_1, τ_2.

Table S5. Comparison of the $\cdot O_2^-$ productions with reported photocatalysts.

Application	Catalyst	Test method	$\cdot O_2^-$ yields (μmol L^{-1})	Irradiation time (min)	Ref.
Pollutant photodegradation	Cu-MoO_3 nanosheet	NBT	14.5	10	[20]
Pollutant photodegradation	Cu-MoO_3 MEM	NBT	29.2	10	[20]
Pollutant photodegradation	CN NT	NBT	9.3	100	[21]
Pollutant photodegradation	N_{2C}V-CN	NBT	23.5	100	[22]
Pollutant photodegradation	BOC	NBT	9.6	30	[23]
Pollutant photodegradation	P-BOC	NBT	36.8	30	[23]
H_2O_2 photosynthesis	CN	NBT	95.2	40	[3]
H_2O_2 photosynthesis	CN-PDA	NBT	209.5	40	[3]

Synergistic pollutant photodegradation and H_2O_2 photosynthesis	CzAQ-3	NBT	4.1	20	[24]
Synergistic pollutant photodegradation and H_2O_2 photosynthesis	CzAQ-2	NBT	12.9	20	[24]
Synergistic pollutant photodegradation and H_2O_2 photosynthesis	CzAQ-1	NBT	53.6	20	[24]
Pollutant photodegradation	Py_{20}-PDI_{19}-PEG_{10}	NBT	22.49	60	This work
Pollutant photodegradation	Cz_{19}-PDI_{18}-PEG_{10}	NBT	29.16	60	This work
Pollutant photodegradation	TPA_{21}-PDI_{19}-PEG_{10}	NBT	62.46	60	This work

Table S6. Comparison of the $^1O_2$ quantum yields with reported photocatalysts.

Application	Catalyst	Test method	$^1O_2$ quantum yields	Benchmark	Ref.
Photocatalytic transformation	PDI-TPE-COF	DPBF	0.45	RB	[25]
Photocatalytic transformation	PDI-DBC-COF	DPBF	0.64	RB	[25]
Photodynamic therapy	TTCF	ABDA	0.18	RB	[26]
Pollutant photodegradation	sp^2c-Py-BPy-COF	DPBF	0.128	RB	[4]

Pollutant photodegradation	Im-Py-BPy-COF	DPBF	0.0108	RB	[4]
Photocatalytic transformation	PDI-based metallacages 4B	ABDA	0.56	RB	[27]
Photocatalytic transformation	PDI-based metallacages 4A	ABDA	0.35	RB	[27]
Photodynamic therapy	NI-Por NPs	ABDA	0.23	Methylene Blue (MB)	[28]
Photodynamic therapy	NI-ZnPor NPs	ABDA	0.21	MB	[28]
Photocatalytic transformation	(R)-DTP-COF-QA	DPBF	0.57	MB	[6]
Photodynamic therapy	Hf-MOF	ABDA	0.19	Protoporphyrin IX (PpIX)	[29]
Photodynamic therapy	Hf-MOL	ABDA	0.78	PpIX	[29]
Pollutant photodegradation	Pc	DPBF	0.558	ZnPc	[30]
Pollutant photodegradation	Pc-MIL-88B (Fe)	DBPF	0.574	ZnPc	[30]
Photocatalytic transformation	PyPor-COF	ABDA	0.778	RB	[31]
Pollutant photodegradation	Py_{20}-PDI_{19}-PEG_{10}	DBPF	0.45	RB	This work
Pollutant photodegradation	Cz_{19}-PDI_{18}-PEG_{10}	DBPF	0.51	RB	This work
Pollutant photodegradation	TPA_{21}-PDI_{19}-PEG_{10}	DBPF	0.59	RB	This work

Table S7. Comparison of the BPA degradation rate constants with reported photocatalysts.

Sample	Light source	$C_{catalyst}$ (g L^{-1})	C_0-BPA (mg L^{-1})	Degradation time (min)	Removal rate (%)	k (min^{-1})	Ref.
TpPdz-COF	10 W LED lamps (λ > 400 nm, 85 mW cm^{-2})	0.25	20	45	97.5	0.07357	[32]
TiO_2/Co-TpYp	50 W LED lamps (λ > 420 nm)	0.2	20	60	94.1	0.0444	[33]
HOFs@Fe^{3+}	300 W Xe lamps	0.5	/	30	100	0.109	[34]
Co-N-C/SA-PDI	300 W Xe lamps (λ > 400 nm, 100 mW cm^{-2})	0.2	10	/	/	0.023	[35]
PDI Micelle	300 W Xe lamps (λ > 420 nm)	0.4	50	480	100	0.00502	[1]
PDI-SiO_2	Xe lamps (λ ≥ 420 nm)	0.5	10	30	100	0.100	[36]
BiOBr/$Bi_4O_5Br_2$/PDI	350W Xe lamps (λ ≥ 420 nm, 103 mW cm^{-2})	0.5	10	80	/	0.0376	[37]
Py_{20}-PDI_{19}-PEG_{10}	300 W Xe lamps (λ > 420 nm, 300 mW cm^{-2})	0.4	50	40	74.4	0.01618	This work
Cz_{19}-PDI_{18}-PEG_{10}	300 W Xe lamps (λ > 420 nm, 300 mW cm^{-2})	0.4	50	40	90.2	0.02039	This work

TPA_{21}-PDI_{19}-PEG_{10}	300 W Xe lamps (λ > 420 nm, 300 mW cm^{-2})	0.4	50	25	100	0.01163	This work

Table S8. Non-linear fitting parameters for adsorption kinetics of pseudo-first-order and pseudo-second-order models.

T	C_0	$q_{e\cdot exp}$	**Pesudo-First-Order Model**			**Pesudo-Second-Order Model**		
(K)	(mg L^{-1})	(mg g^{-1})	k_1 (s^{-1})	$q_{e\cdot cal}$ (mg g^{-1})	R^2	k_2 (g mg^{-1} s^{-1})	$q_{e\cdot cal}$ (mg g^{-1})	R^2
298	10	18.69	0.516	18.15	0.99428	0.012	18.47	0.99809
	30	49.27	0.430	47.70	0.99527	0.028	48.72	0.99900
	50	81.22	0.608	79.23	0.99713	0.036	80.20	0.99911
	100	134.52	0.544	130.53	0.99602	0.017	132.48	0.99852
	150	185.36	0.697	179.68	0.99711	0.023	181.31	0.99827
	200	229.00	0.608	225.49	0.99842	0.014	227.90	0.99967
273	10	19.84	0.697	19.46	0.99892	0.238	19.61	0.99965
	30	53.47	0.796	52.73	0.99946	0.153	52.96	0.99967
	50	89.38	0.530	87.76	0.99347	0.021	89.40	0.99823
	100	157.59	0.510	154.16	0.99788	0.013	156.49	0.99983
	150	235.78	0.658	233.69	0.99952	0.019	235.39	0.99992
	200	292.16	0.601	290.03	0.99884	0.011	293.03	0.99990
323	10	13.77	0.255	12.79	0.95329	0.035	13.45	0.98775
	30	38.33	0.295	35.36	0.95842	0.015	37.03	0.98710
	50	62.44	0.567	62.74	0.99583	0.037	63.67	0.99863
	100	107.75	0.316	102.66	0.97160	0.006	106.91	0.99256
	150	137.65	0.336	133.34	0.98703	0.006	137.84	0.99885
	200	173.95	0.484	162.78	0.98551	0.009	166.61	0.99287

Table S9. Non-linear fitting parameters at different temperatures (C_0=50 mg L^{-1}).

T	$q_{e\cdot exp}$	**Pesudo-Second-Order Model**			**Activation Energy**
(K)	(mg g^{-1})	k_2 (s^{-1})	$q_{e\cdot cal}$ (mg g^{-1})	R^2	E_a (kJ mol^{-1})
273	89.38	0.02092	89.40	0.99823	8.595
298	81.22	0.03604	80.20	0.99911	
323	62.44	0.03711	63.67	0.99863	

Table S10. Non-linear fitting parameters of adsorption isotherms of Langmuir and Freundlich models.

T	Langmuir isotherm			Freundlich isotherm			Thermodynamic parameters		
(K)	q_m (mg g^{-1})	k_L (L mg^{-1})	R^2	k_F (mg^{1-n} L^n g^{-1})	n	R^2	ΔG (kJ mol^{-1})	ΔH (kJ mol^{-1})	ΔS (J mol^{-1} K^{-1})
273	661.81	0.00951	0.99023	12.40	1.390	0.99940	-5.109	-20.910	-55.586
298	372.93	0.01334	0.98710	12.59	1.620	0.99973	-4.931		
323	276.99	0.01108	0.99709	9.16	1.675	0.99862	-2.946		

Reference

[1] G. Wang, H. Li, N. J. Li, D. Y. Chen, J. H. He, Q. F. Xu, J. M. Lu, *Angew Chem Int Edit* **2022**, *61* (40), https://doi.org/ARTN e202210619
10.1002/anie.202210619.

[2] J. Gao, L. J. Yang, G. Wang, C. F. Xie, H. Yin, H. Li, J. M. Lu, *Small* **2025**, *21* (9), https://doi.org/ARTN 2410805
10.1002/smll.202410805.

[3] Y. Deng, W. Liu, R. Xu, R. Gao, N. Huang, Y. Zheng, Y. Huang, H. Li, X. Y. Kong, L. Ye, *Angewandte Chemie International Edition* **2024**, *63* (14), e202319216, https://doi.org/https://doi.org/10.1002/anie.202319216.

[4] S. Guo, K. Zhao, L. Liang, Z. Li, B. Han, X. Ou, S. Yao, Z. Lin, Z. Dong, Y. Liu, L. Ye, B. Weng, Y. Cai, Z. Yang, *Angewandte Chemie International Edition* **2025**, *64* (31), e202509141, https://doi.org/https://doi.org/10.1002/anie.202509141.

[5] X. Zhan, Y. Jin, C. Qu, H. Liu, R. Jiang, Q. Zhi, D. Qi, K. Wang, B. Han, H. Pan, J. Jiang, *Advanced Functional Materials* **2025**, *35* (8), 2415629, https://doi.org/https://doi.org/10.1002/adfm.202415629.

[6] X. Kan, J.-C. Wang, Z. Chen, J.-Q. Du, J.-L. Kan, W.-Y. Li, Y.-B. Dong, *Journal of the American Chemical Society* **2022**, *144* (15), 6681, https://doi.org/10.1021/jacs.2c01186.

[7] P. J. Stephens, F. J. Devlin, C. F. Chabalowski, M. J. Frisch, *The Journal of Physical Chemistry* **1994**, *98* (45), 11623, https://doi.org/10.1021/j100096a001.

[8] a) W. J. Hehre, R. Ditchfield, J. A. Pople, *The Journal of Chemical Physics* **1972**, *56* (5), 2257, https://doi.org/10.1063/1.1677527; b) M. M. Francl, W. J. Pietro, W. J. Hehre, J. S. Binkley, M. S. Gordon, D. J. DeFrees, J. A. Pople, *The Journal of Chemical Physics* **1982**, *77* (7), 3654, https://doi.org/10.1063/1.444267.

[9] P. C. Hariharan, J. A. Pople, *Theoretica chimica acta* **1973**, *28* (3), 213, https://doi.org/10.1007/BF00533485.

[10] T. Yanai, D. P. Tew, N. C. Handy, *Chemical Physics Letters* **2004**, *393* (1), 51, https://doi.org/https://doi.org/10.1016/j.cplett.2004.06.011.

[11] a) M. J. Frisch, J. A. Pople, J. S. Binkley, *The Journal of Chemical Physics* **1984**, *80* (7), 3265, https://doi.org/10.1063/1.447079; b) R. Krishnan, J. S. Binkley, R. Seeger, J. A. Pople, *The Journal of Chemical Physics* **1980**, *72* (1), 650, https://doi.org/10.1063/1.438955.

[12] S. P. D. Monte Blanco, F. B. Scheufele, A. N. Módenes, F. R. Espinoza-Quiñones, P. Marin, A. D. Kroumov, C. E. Borba, *Chemical Engineering Journal* **2017**, *307*, 466, https://doi.org/https://doi.org/10.1016/j.cej.2016.08.104.

[13] M. Chaturvedi, N. Kaur, C. Jeyaseelan, M. Sillanpää, S. A. Farraj, S. Sharma, *Environmental Research* **2024**, *255*, 119192, https://doi.org/https://doi.org/10.1016/j.envres.2024.119192.

[14] N. Sakkayawong, P. Thiravetyan, W. Nakbanpote, *Journal of Colloid and Interface Science* **2005**, *286* (1), 36, https://doi.org/https://doi.org/10.1016/j.jcis.2005.01.020.

[15] R. Chen, Z. Li, G. Cheng, M. Zhang, G. Huo, Y. Liu, G. Zhu, *Journal of Environmental Management* **2026**, *401*, 128865, https://doi.org/https://doi.org/10.1016/j.jenvman.2026.128865.

[16] N. Zhang, Y. Li, M. Liu, M. Hu, H. Wang, W. Ma, M. Lu, *Journal of Hazardous Materials* **2025**, *493*, 138382, https://doi.org/https://doi.org/10.1016/j.jhazmat.2025.138382.

[17] X. Jiang, J. Zhao, T. Yu, X. He, L. Chen, Y. Zhang, *Journal of Hazardous Materials* **2025**, *494*, 138616, https://doi.org/https://doi.org/10.1016/j.jhazmat.2025.138616.

[18] Y. Ding, P. Zhou, H. Tang, *Chem. Eng. J.* **2016**, *291*, 149, https://doi.org/https://doi.org/10.1016/j.cej.2016.01.105.

[19] a) G. Ferrara, E. Loffredo, N. Senesi, *Planta* **2006**, *223* (5), 910, https://doi.org/10.1007/s00425-005-0147-2; b) H. Qu, D. An, G. Li, W. Xu, C. Ma, H. Zhang, E. Bahojb Noruzi, J. Cheng, C. Zhou, G. Periyasami, H. Li, *ACS Applied Materials & Interfaces* **2024**, *16* (45), 62925, https://doi.org/10.1021/acsami.4c12738.

[20] J. Ye, C. Yu, J. Dai, L. Li, R. Zhang, J. Pan, W. Xue, Y. Jiang, J. Zhao, *Advanced Materials* **2026**, *38* (5), e14600, https://doi.org/https://doi.org/10.1002/adma.202514600.

[21] X. Zhang, X. Li, P. Yu, Y. Yu, X. Fan, J. Zhang, Y. Yu, H. Zheng, Y. Sun, *Journal of Hazardous Materials* **2023**, *456*, 131715, https://doi.org/https://doi.org/10.1016/j.jhazmat.2023.131715.

[22] J. Yue, H. Yang, C. Liu, S. Wang, L. Wang, *Journal of Colloid and Interface Science* **2024**, *673*, 475, https://doi.org/https://doi.org/10.1016/j.jcis.2024.06.104.

[23] H. Xu, X. Liu, H. Li, L. Zhang, *Applied Catalysis B: Environmental* **2022**, *314*, 121520, https://doi.org/https://doi.org/10.1016/j.apcatb.2022.121520.

[24] Y. Cheng, Y.-X. Ye, Y. Huang, H. Yan, L. Zhang, F. Zhu, G. Ouyang, *Applied Catalysis B: Environment and Energy* **2024**, *355*, 124166, https://doi.org/https://doi.org/10.1016/j.apcatb.2024.124166.

[25] D. Cao, C. Gong, Y. Han, C. Zhu, Y. Ma, Q. Xia, Y. Peng, G. Yuan, *Angewandte Chemie International Edition* **2025**, *n/a* (n/a), e202516908, https://doi.org/https://doi.org/10.1002/anie.202516908.

[26] Y. Choi, Y. Lee, D. Kang, Y. Kim, *Chemical Communications* **2025**, *61* (89), 17412, https://doi.org/10.1039/D5CC04669F.

[27] Y. Hou, Z. Zhang, L. Ma, R. Shi, S. Ling, X. Li, G. He, M. Zhang, *CCS Chemistry* **2021**, *4* (8), 2604, https://doi.org/10.31635/ccschem.021.202101382.

[28] M. Yang, S. Cao, X. Sun, H. Su, H. Li, G. Liu, X. Luo, F. Wu, *Bioconjugate Chemistry* **2020**, *31* (3), 663, https://doi.org/10.1021/acs.bioconjchem.9b00819.

[29] T. Luo, Y. Fan, J. Mao, E. Yuan, E. You, Z. Xu, W. Lin, *Journal of the American Chemical Society* **2022**, *144* (12), 5241, https://doi.org/10.1021/jacs.2c00384.

[30] Y.-L. Z. Wen-Bo ZHAO, Guang-Yu XU, Xiao JIA, Jin-Ping XUE, *Chinese Journal of Structural Chemistry* **2021**, *40* (8), 1012, https://doi.org/10.14102/j.cnki.0254-5861.2011-3104.

[31] K.-K. Niu, T.-X. Luan, J. Cui, H. Liu, L.-B. Xing, P.-Z. Li, *ACS Catalysis* **2024**, *14* (4), 2631, https://doi.org/10.1021/acscatal.3c05454.

[32] Y. Luo, X. Wang, L. Ding, C. Lu, L. Liu, F. Deng, W. Ren, P. Shao, J. Zou, X. Luo, *Applied Catalysis B: Environment and Energy* **2026**, *385*, 126251, https://doi.org/https://doi.org/10.1016/j.apcatb.2025.126251.

[33] D. Gu, W. Zhong, C. Li, Y. Xia, Q. Zhu, *Chemical Engineering Journal* **2025**, *519*, 165169, https://doi.org/https://doi.org/10.1016/j.cej.2025.165169.

[34] L. Yang, J. Yuan, G. Wang, Q. Cao, C. Zhang, M. Li, J. Shao, Y. Xu, H. Li, J. Lu, *Advanced Functional Materials* **2023**, *33* (28), 2300954, https://doi.org/https://doi.org/10.1002/adfm.202300954.

[35] X. Liu, Y. Zhang, P. Sun, F. He, Y. Wu, S. Wang, S. Wang, J. Zhang, *Angewandte Chemie International Edition* **2025**, *64* (28), e202507028, https://doi.org/https://doi.org/10.1002/anie.202507028.

[36] W. Li, H. Zhang, S. Huang, J. Xu, L. Liu, J. Li, J. Jing, Y. Zhu, *Applied Catalysis B: Environmental* **2024**, *340*, 123262, https://doi.org/https://doi.org/10.1016/j.apcatb.2023.123262.

[37] H. Wang, Y. Zhou, J. Wang, A. Li, P. François-Xavier Corvini, *Chemical Engineering Journal* **2022**, *433*, 133622, https://doi.org/https://doi.org/10.1016/j.cej.2021.133622.
